# First Trimester Tetracycline Exposure and Risk of Major Congenital Malformations

**DOI:** 10.1001/jamanetworkopen.2024.45055

**Published:** 2024-11-14

**Authors:** Aya Olivia Nakitanda, Ingvild Odsbu, Carolyn E. Cesta, Laura Pazzagli, Björn Pasternak

**Affiliations:** 1 Centre for Pharmacoepidemiology, Department of Medicine Solna, Karolinska Institutet, Stockholm, Sweden; 2 Department of Chronic Diseases, Norwegian Institute of Public Health, Oslo, Norway; 3 Clinical Epidemiology Division, Department of Medicine Solna, Karolinska Institutet, Stockholm, Sweden; 4 Department of Epidemiology Research, Statens Serum Institut, Copenhagen, Denmark

## Abstract

**Importance:**

Safety data on tetracycline antibiotic use during pregnancy are limited.

**Objective:**

To investigate the association between first trimester exposure to tetracyclines and the risk of major congenital malformations (MCMs).

**Design, Setting, and Participants:**

This cohort study used data from nationwide registers for singleton infants live-born in Sweden from July 1, 2006, to December 31, 2018, with follow-up through December 2019. Tetracycline-exposed and unexposed infants were matched on propensity scores (ratio 1:10). Data analysis was performed from June 2023 to May 2024.

**Exposure:**

First trimester exposure to tetracyclines determined from maternal prescription fills, compared with no exposure.

**Main Outcomes and Measures:**

The primary outcome was any MCM diagnosed in the first year of life; secondary outcomes were 12 major malformation organ system subgroups and 16 individual malformations selected on the basis of prior safety signals and/or fulfillment of prespecified statistical power criteria. Malformations were identified by *International Statistical Classification of Diseases and Related Health Problems, Tenth Revision (ICD-10)* codes aligned to the European Surveillance of Congenital Anomalies (EUROCAT) classification. Log binomial regression was used to estimate relative risks (RRs) with 95% CIs.

**Results:**

From a source cohort of 1 245 889 eligible infants (640 892 male [51.4%]), a propensity score–matched analytical cohort of 69 656 infants (35 903 male [51.5%]) was generated. Of 6340 infants exposed to tetracyclines during the first trimester (3325 male [52.4%]), 252 received a diagnosis of any MCM (39.75 cases per 1000 infants; 95% CI, 35.14-44.93 cases per 1000 infants) compared with 2454 of 63 316 unexposed infants (38.76 cases per 1000 infants; 95% CI, 37.27-40.30 cases per 1000 infants). Tetracycline exposure was not associated with any MCM (RR, 1.03; 95% CI, 0.90-1.16). The RRs for specific tetracycline substances were 1.07 (95% CI, 0.93-1.23) for doxycycline, 0.83 (95% CI, 0.60-1.15) for lymecycline, and 0.78 (95% CI, 0.32-1.92) for tetracycline-oxytetracycline. There was no increased risk for 10 of 12 malformation subgroups or for any of the 16 individual malformations analyzed. The higher RRs in the main analysis for nervous system anomalies (1.92; 95% CI, 0.98-3.78) and eye anomalies (1.76; 95% CI, 1.07-2.91) were attenuated in a sensitivity analysis with follow-up extended to age 3 years (nervous system anomalies, RR, 1.08; 95% CI, 0.52-2.24; eye anomalies, RR, 1.42; 95% CI, 0.88-2.29).

**Conclusions and Relevance:**

In this cohort study, first trimester tetracycline exposure was not associated with increased risks of any MCMs. Additional studies are needed to rule out potential risks owing to power limitations for several MCM subgroups and individual malformations.

## Key Points

**Question:**

Is first trimester exposure to tetracycline antibiotics associated with increased risks of major congenital malformations?

**Findings:**

In this Swedish population-based cohort study of 6340 infants exposed to tetracycline antibiotics during the first trimester, the risks of major congenital malformations overall, major congenital malformation subgroups, and selected individual malformations were not higher than those among propensity score–matched, unexposed controls.

**Meaning:**

Although first trimester tetracycline exposure was not associated with increased risks of major congenital malformations, yet larger studies are needed to rule out risks of several malformation subgroups and individual malformations.

## Teaser

This cohort study examines the association between first trimester exposure to tetracyclines and the risk of major congenital malformations among infants in Sweden.

## Introduction

Tetracycline antibiotics exhibit activity against both gram-positive and gram-negative bacteria, as well as drug-resistant bacteria and parasites, and are widely used for common bacterial infections, sexually transmitted infections, skin infections including acne, and atypical respiratory infections.^1,2^ In pregnancy, tetracycline use is most prevalent during the first trimester, with doxycycline most frequently prescribed.^3,4,5,6^ Indeed, a recent study^6^ found doxycycline to be the ninth most commonly used prescription drug overall in the first trimester, used by 3.8% of commercially insured women in the US.

Tetracyclines are, however, not recommended for use during pregnancy, especially from the second trimester onward. They are known to cross the placenta and have historically been implicated in the inhibition of skeletal development, dental hypoplasia, and irreversible discoloration following later pregnancy exposure, on the basis of evidence primarily from older-generation, now infrequently used substances such as tetracycline and oxytetracycline.^7^ Yet, concerns pertaining to early pregnancy use remain, owing to the limited and inconsistent epidemiological findings on other congenital malformations and specific tetracycline substances.^8,9,10^ More recent population-based studies have not definitively ruled out risks of specific malformations reported in earlier research,^11,12,13^ as the majority were constrained by small samples of tetracycline-exposed individuals (1694 in the largest cohort study to date),^14^ resulting in limited statistical power to discern associations with individual malformations.^5,14,15,16,17^

Safety information is crucial to guide prescribing and communicate risks in clinical situations that necessitate tetracycline use during pregnancy. We aimed to investigate the association between first trimester tetracycline exposure and risk of major congenital malformations (MCMs).

## Methods

### Study Design

We performed a population-based cohort study using individually linked data from nationwide Swedish health and population registers (eTables 1 and 2 in Supplement 1). The study was approved by the Regional Ethics Committee in Stockholm, and followed the Strengthening the Reporting of Observational Studies in Epidemiology (STROBE) reporting guidelines for observational studies. Informed consent for participation in large-scale register-based studies is not required in Sweden.

### Study Population

From the Medical Birth Register,^18^ we selected all singletons born alive in Sweden between July 1, 2006, and December 31, 2018, and excluded those with implausible or missing gestational age at birth; with exposure to known teratogens during the first trimester; whose mothers did not reside in Sweden continuously during the 1-year period prior to pregnancy until birth; who themselves did not reside continuously in Sweden during the first year of life; or who, during the first year of life, received diagnoses of teratogenic syndromes of known causes, chromosomal aberrations, genetic disorders, or viral infections potentially resulting in malformations (eTable 1 in Supplement 1). Infants born to mothers with at least 1 filled prescription for tetracycline 90 days prior to pregnancy, but not during the first trimester, were excluded to preclude exposure misclassification since drugs collected during 90 days prior to pregnancy could have been consumed during first trimester.

### Exposure

The Prescribed Drug Register contains records of all prescriptions filled at pharmacies across Sweden,^19^ and we determined the exposure status of the study population from the Anatomical Therapeutic Chemical (ATC) code J01A (systemic tetracyclines) and prescription fill dates. First trimester exposure was defined as having had at least 1 prescription filled by the mother between the first day of the last menstrual period and 97 days of gestation. The last menstrual period was calculated as the date of birth minus gestational age at birth in days. Gestational age is ultrasonography-estimated around 18 weeks of gestation, when at least 95% of pregnant women in Sweden undergo routine ultrasonography.^18^ Infants born to mothers without any prescription filled for tetracyclines during the first trimester were considered unexposed.

### Outcomes

MCMs are inborn structural changes that have significant medical, surgical, social, or cosmetic consequences and typically require medical intervention.^20^ These exclude minor anomalies that pose little or no substantial health problems and tend to have limited consequences.^21^ MCMs were defined by *International Statistical Classification of Diseases and Related Health Problems, Tenth Revision* Swedish diagnostic codes aligned to the European Surveillance of Congenital Anomalies (EUROCAT) Guidelines version 1.5 (eTable 2 in Supplement 1).^21^ The primary outcome of any MCM in the first year of life was captured from any inpatient or specialist outpatient care diagnoses recorded in the National Patient Register^22^ or from any underlying or contributing causes of death in the Cause of Death Register.^23^ Follow-up data were available through December 2019, allowing a 1-year follow-up for all included infants. As secondary outcomes, we investigated all EUROCAT MCM organ system subgroups (eTable 2 in Supplement 1)^21^; selected subgroups and individual MCMs previously reported to be associated with tetracyclines, including cardiac anomalies,^12,15^ ventricular and/or atrial septal defects,^15^ cleft lip with or without cleft palate,^13,16^ cleft palate,^11,12^ neural tube defects,^12^ esophageal atresia or stenosis,^13^ and limb reduction defects^7^; as well as individual MCMs for which a 2.5-fold relative risk (RR) increase was detectable at 90% power (5% α-level). Preliminary analysis showed a potential analytical cohort of 6341 exposed and 63 410 unexposed individuals (matching ratio 1:10), which would require a minimal prevalence of 0.91 malformation cases per 1000 unexposed individuals. We then assessed the prevalence of all MCM categories from the EUROCAT classification in the source cohort, generating 8 additional individual MCMs that fulfilled the power criterion (eTable 3 in Supplement 1). Power calculations were performed using OpenEpi.^24^

### Statistical Analysis

We used propensity score matching to account for confounding.^25^ Propensity scores were calculated using logistic regression as the estimated probability of tetracycline exposure given selected sociodemographic, obstetric, lifestyle, and medical characteristics suspected to be associated with tetracycline exposure and/or MCMs (eTable 4 in Supplement 1). Nearest neighbor matching algorithm (caliper width 0.2 SD) was used to match each tetracycline-exposed infant with up to 10 unexposed infants by propensity score without replacement. Covariates were considered balanced when the absolute standardized mean difference was less than 0.1. Missing values were treated as a variable category.

The prevalence of any MCMs, MCM subgroups, and individual MCMs per 1000 infants with 95% CI were calculated for tetracycline-exposed and unexposed groups. RRs with 95% CIs for all outcomes were estimated using log binomial regression comparing exposed with unexposed groups.^26^ Cluster-robust variance estimator was used to account for correlations between infants born from the same mother. No adjustments were made for multiple comparisons to avoid missing potential safety signals.^27^ Statistical analyses were performed using R statistical software version 4.3.2 (R Project for Statistical Computing).^28^ Data analysis was performed from June 2023 to May 2024.

#### Sensitivity Analyses

To assess the robustness of our results, we conducted 3 sensitivity analyses. First, the outcome definition was made stricter such that at least 1 inpatient diagnosis, 1 cause of death diagnosis or 2 (instead of 1) outpatient diagnoses in specialist care were required. Second, the follow-up time was extended to 3 years from birth to accommodate longer detection time. A separate propensity score–matched subcohort was constructed with inclusion of births from July 2006, through December 2016, which allowed for 3 years of follow-up. Third, covariates used in the propensity score estimation with missing values were imputed using multiple imputation by chained equations. Propensity score matching and regression analyses were performed within each of the 5 multiply imputed datasets generated on the basis of 10 iterations, subsequently pooling the effect estimates. All sensitivity analyses were applied to any MCM and those MCMs for which an increased risk was observed in the main analysis. The analysis with a 3-year follow-up was conducted for MCM subgroups at risk of underdetection with a 1-year follow-up. These included nervous system anomalies; eye anomalies; ear, face, and neck anomalies; genital anomalies; and other anomalies.^29^

#### Supplemental Analyses

Potential differential associations by specific tetracycline substances and duration of treatment were assessed, from subcohorts of exposed infants with maternal prescription fills for only 1 tetracycline substance and 1 prescription for oral formulations filled during the first trimester, respectively. Distinct propensity scores were calculated for each of these analyses. Short-term and long-term user groups were determined from the volume of active substance in the sole prescription filled during the first trimester, with short-term treatment limits set at 30 defined daily doses (DDDs) for doxycycline (given that daily doses of up to 2 DDDs are prescribed for some infections) and 15 DDDs for lymecycline, tetracycline, and oxytetracycline.

## Results

### Cohort

**Figure 1.**
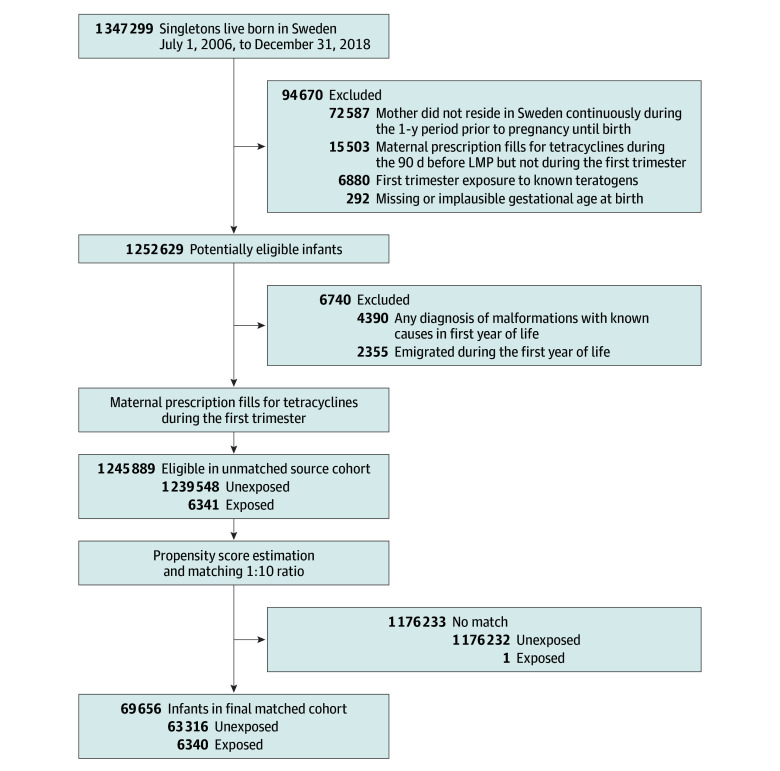
Flowchart Depicting Cohort Selection LMP indicates last menstrual period.

Of 1 245 889 eligible infants (640 892 male [51.4%]) included in the source cohort, 6341 (0.5%) were exposed to tetracyclines during the first trimester (Figure 1 and Table 1). Compared with unexposed infants, infants exposed to tetracyclines were more likely to be born in earlier calendar years. Compared with mothers of unexposed infants, mothers of tetracycline-exposed infants were more often in the youngest and oldest age strata, Swedish-born, had attained lower levels of education, had diagnosed substance use disorders, smoked during early pregnancy, used prescription drugs, and utilized health care prior to pregnancy in higher proportions (Table 1). Following propensity score matching, the analytical cohort included 69 656 infants (35 903 male [51.5%]); 6340 were tetracycline exposed and 63 316 were unexposed, with the groups balanced on all covariates (Table 1 and eFigure 1A in Supplement 1). Among the 6340 exposed infants (3325 male [52.4%]), 4962 (78.3%) were exposed to doxycycline, 1199 (18.9%) to lymecycline, 180 (2.8%) to tetracycline, and 13 (0.2%) to oxytetracycline.

**Table 1.** Characteristics of Study Population Before and After Propensity Score Matching

Characteristic	Infants, No. (%)			
	Before propensity score matching		After propensity score matching	
	Unexposed (n = 1 239 548)	Tetracycline exposed (n = 6341)	Unexposed (n = 63 316)	Tetracycline exposed (n = 6340)
Calendar year of birth				
2006-2009	329 596 (26.6)	2062 (32.5)	21 100 (33.3)	2061 (32.5)
2010-2012	299 091 (24.1)	1695 (26.7)	16 711 (26.4)	1695 (26.7)
2013-2015	302 475 (24.4)	1424 (22.5)	13 779 (21.8)	1424 (22.5)
2016-2018	308 386 (24.9)	1160 (18.3)	11 726 (18.5)	1160 (18.3)
Maternal age at delivery, y				
<20	14 506 (1.2)	160 (2.5)	1319 (2.1)	159 (2.5)
20-24	146 953 (11.9)	906 (14.3)	8897 (14.1)	906 (14.3)
25-29	374 336 (30.2)	1704 (26.9)	17 479 (27.6)	1704 (26.9)
30-34	429 580 (34.7)	1968 (31.0)	20 001 (31.6)	1968 (31.0)
35-39	224 244 (18.1)	1250 (19.7)	12 623 (19.9)	1250 (19.7)
40-44	47 423 (3.8)	329 (5.2)	2845 (4.5)	329 (5.2)
≥45	2504 (0.2)	24 (0.4)	152 (0.2)	24 (0.4)
Missing	<3 (<0.1)	0	0	0
Maternal country of birth				
Sweden	972 421 (78.4)	5 116 (80.7)	51 056 (80.6)	5115 (80.7)
Other Nordic country	14 928 (1.2)	58 (0.9)	610 (1.0)	58 (0.9)
Others	252 199 (20.3)	1167 (18.4)	11 650 (18.4)	1167 (18.4)
Maternal education				
Compulsory	126 945 (10.2)	931 (14.7)	8903 (14.1)	931 (14.7)
Pre-university	615 388 (49.6)	3249 (51.2)	32 539 (51.4)	3249 (51.2)
Tertiary	482 323 (38.9)	2080 (32.8)	21 343 (33.7)	2080 (32.8)
Missing	14 892 (1.2)	81 (1.3)	531 (0.8)	80 (1.3)
Cohabiting partnership	1 107 598 (89.4)	5316 (83.8)	52 878 (83.5)	5316 (83.8)
Body mass index in early pregnancy^a^				
<18.5, Underweight	27 683 (2.2)	150 (2.4)	1483 (2.3)	150 (2.4)
18.5 to <25.0, Normal	684 302 (55.2)	3366 (53.1)	33 700 (53.2)	3365 (53.1)
25.0 to <30.0, Pre-obesity	294 010 (23.7)	1480 (23.3)	15 041 (23.8)	1480 (23.3)
30.0 to <35.0, Obesity class I	106 036 (8.6)	574 (9.1)	5830 (9.2)	574 (9.1)
35.0 to <40.0, Obesity class II	33 705 (2.7)	222 (3.5)	1857 (2.9)	222 (3.5)
≥40.0, Obesity class III	12 123 (1.0)	91 (1.4)	774 (1.2)	91 (1.4)
Missing	81 689 (6.6)	458 (7.2)	4631 (7.3)	458 (7.2)
Conception by assisted reproductive technology	64 347 (5.2)	329 (5.2)	3220 (5.1)	329 (5.2)
Any major congenital malformation in previous births (among parous)	30 795 (4.4)	147 (4.3)	1396 (4.0)	147 (4.3)
Other chronic conditions				
Preexisting diabetes	10 138 (0.8)	51 (0.8)	500 (0.8)	51 (0.8)
Alcohol use disorder	2866 (0.2)	46 (0.7)	381 (0.6)	45 (0.7)
Other substance use disorder	3281 (0.3)	63 (1.0)	537 (0.8)	63 (1.0)
Maternal smoking in early pregnancy				
No	1 125 283 (90.8)	5471 (86.3)	55 796 (88.1)	5470 (86.3)
Yes	68 713 (5.5)	632 (10.0)	5084 (8.0)	632 (10.0)
Missing	45 552 (3.7)	238 (3.8)	2436 (3.8)	238 (3.8)
Health care utilization				
Prescription fills in past 3 mo (unique Anatomical Therapeutic Chemical second level), No.				
0-1	1 030 861 (83.2)	4287 (67.6)	42 548 (67.2)	4287 (67.6)
2-4	190 565 (15.4)	1721 (27.1)	17 736 (28.0)	1721 (27.1)
>4	18 122 (1.5)	333 (5.3)	3032 (4.8)	332 (5.2)
Specialist outpatient visits in past year, No.				
0	596 784 (48.1)	1995 (31.5)	18 938 (29.9)	1995 (31.5)
1-2	249 532 (20.1)	1762 (27.8)	17 791 (28.1)	1762 (27.8)
>2	393 232 (31.7)	2584 (40.8)	26 587 (42)	2583 (40.7)
Hospitalizations in past year	172 020 (13.9)	857 (13.5)	8425 (13.3)	856 (13.5)
Other prescription drug use in past year				
Antihypertensives	9730 (0.8)	95 (1.5)	924 (1.5)	95 (1.5)
Antidiabetics	9831 (0.8)	46 (0.7)	453 (0.7)	46 (0.7)
Antimycotics	16 636 (1.3)	263 (4.1)	2564 (4.0)	262 (4.1)
Antivirals	12 951 (1.0)	146 (2.3)	1369 (2.2)	145 (2.3)
Antidepressants	60 159 (4.9)	580 (9.1)	5665 (8.9)	580 (9.1)
Antipsychotics	4430 (0.4)	55 (0.9)	511 (0.8)	55 (0.9)
Benzodiazepines	21 272 (1.7)	289 (4.6)	2580 (4.1)	289 (4.6)
Antiepileptics	5827 (0.5)	74 (1.2)	657 (1.0)	74 (1.2)
Opioids	34 335 (2.8)	406 (6.4)	3797 (6.0)	406 (6.4)
Nonsteroidal anti-inflammatory drugs	72 527 (5.9)	829 (13.1)	8295 (13.1)	828 (13.1)
Systemic corticosteroids	18 076 (1.5)	344 (5.4)	3056 (4.8)	344 (5.4)
Oral contraceptives	57 801 (4.7)	410 (6.5)	3985 (6.3)	409 (6.5)
Ovulation induction	62 211 (5.0)	358 (5.6)	3547 (5.6)	358 (5.6)
Potentially teratogenic drugs	11 646 (0.9)	318 (5.0)	2825 (4.5)	317 (5.0)
Prescription folate	22 789 (1.8)	211 (3.3)	2065 (3.3)	211 (3.3)
Self-reported folate use	242 413 (19.6)	1141 (18.0)	10 870 (17.2)	1141 (18.0)
Other antibiotic use during first trimester	96 398 (7.8)	1258 (19.8)	12 928 (20.4)	1257 (19.8)
Hospitalization for infection during first trimester	1821 (0.1)	72 (1.1)	457 (0.7)	71 (1.1)

^a^ Body mass index is calculated as weight in kilograms divided by height in meters squared.

### Main Analysis

**Figure 2.**
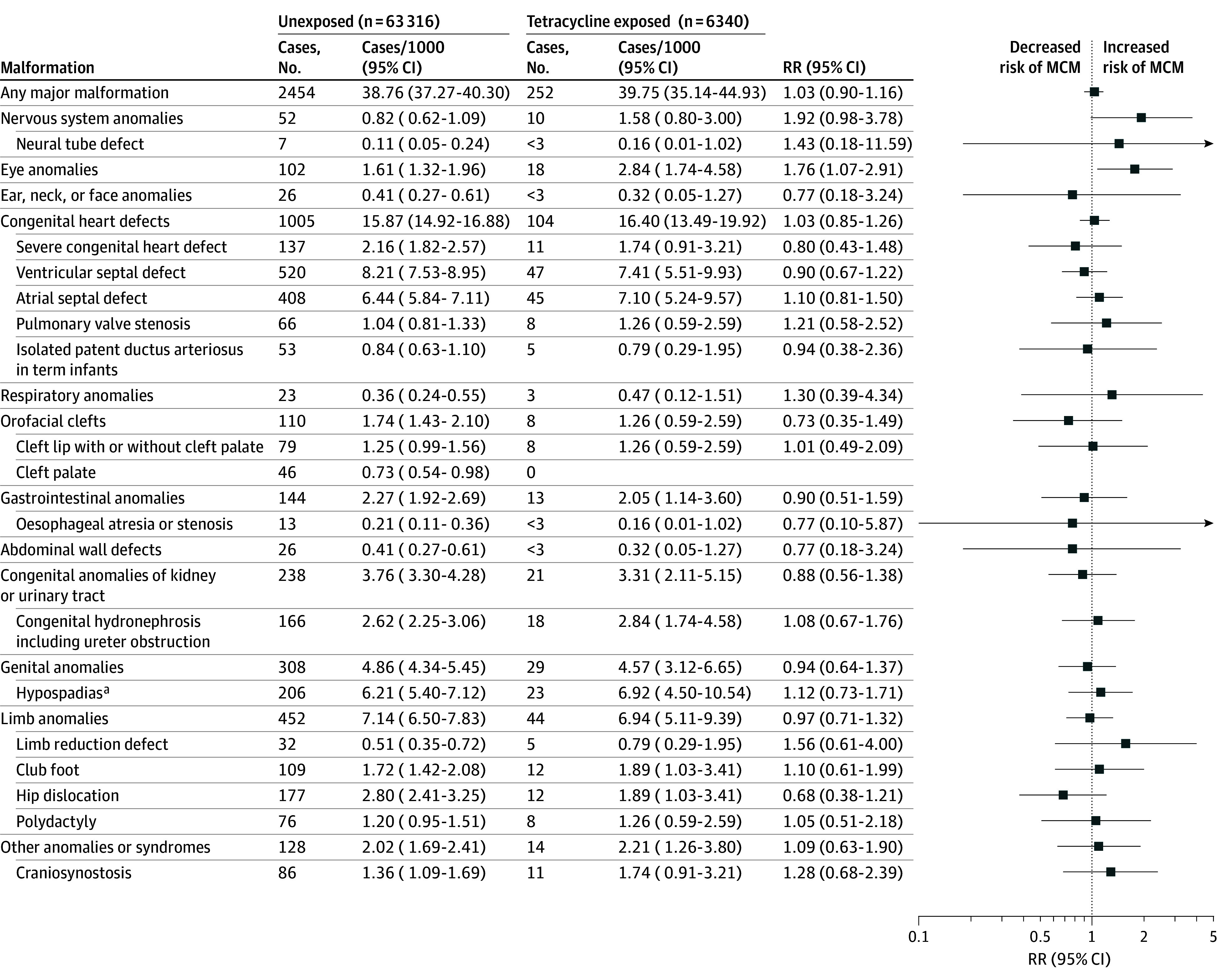
Association Between First Trimester Tetracycline Exposure and Major Congenital Malformations (MCMs) RR indicates relative risk. ^a^The male population includes 33 199 unexposed infants and 3323 exposed infants.

The prevalence of any MCMs among tetracycline-exposed infants was 39.75 cases per 1000 infants (95% CI, 35.14-44.93 cases per 1000 infants; 252 infants) and that among unexposed infants was 38.76 cases per 1000 infants (95% CI, 37.27-40.30 cases per 1000 infants; 2454 infants) (Figure 2). The risk of any MCM did not differ between the groups (RR, 1.03; 95% CI, 0.90-1.16). Tetracycline exposure was not associated with increased risks for 10 of 12 MCM subgroups, or with any of the 16 individual MCMs analyzed. Higher RRs were observed for 2 of the MCM subgroups: nervous system anomalies (RR, 1.92; 95% CI, 0.98-3.78) and eye anomalies (RR, 1.76; 95% CI, 1.07-2.91).

### Sensitivity Analyses

**Figure 3.**
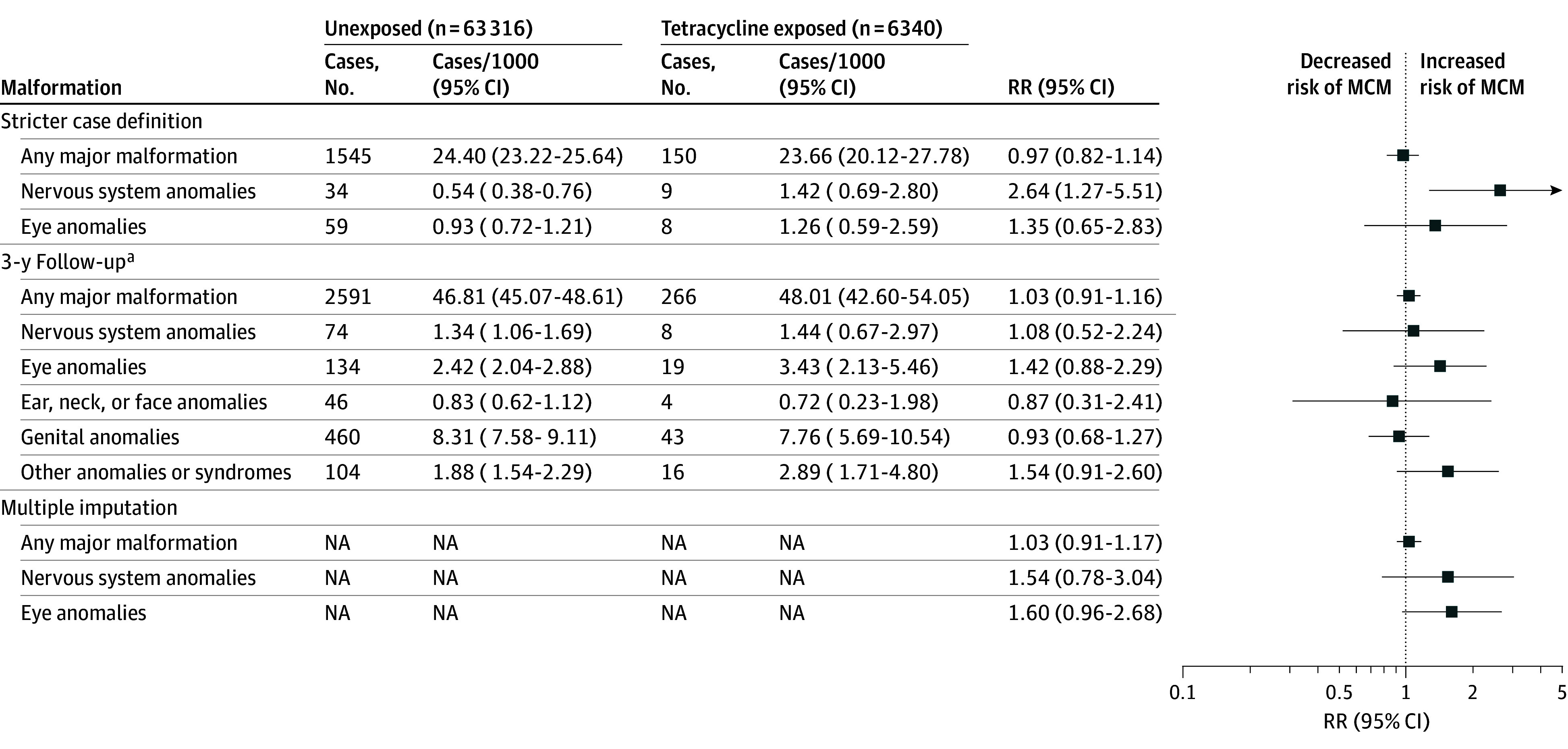
Sensitivity Analyses on Association Between First Trimester Tetracycline Exposure and Major Congenital Malformations (MCMs) With a Stricter Case Definition, Follow-Up Extended to Age 3 Years and Multiply Imputed Data NA indicates not applicable; RR, relative risk. ^a^There were 55 353 unexposed infants and 5541 exposed infants.

In analyses that applied a stricter outcome definition requiring at least 2 specialist outpatient care visits with MCM diagnosis, the prevalences of nervous system anomalies and eye anomalies were lower than in the main analysis (Figure 3). The RRs were 2.64 (95% CI, 1.27-5.51) for nervous system anomalies and 1.35 (95% CI, 0.65-2.83) for eye anomalies. With an extended follow-up period of 3 years from birth, the prevalence of nervous system anomalies in the unexposed group was 1.6-fold higher than in the main analysis with 1-year follow-up, whereas that of eye anomalies was 1.5-fold higher (Figure 3). The RRs in this analysis were 1.08 (95% CI, 0.52-2.24) for nervous system anomalies and 1.42 (95% CI, 0.88-2.29) for eye anomalies. Analyses with multiply imputed data yielded RRs of 1.54 (95% CI, 0.78-3.04) for nervous system anomalies and 1.60 (95% CI, 0.96-2.68) for eye anomalies (Figure 3). Unadjusted and sensitivity analyses using propensity scores based on covariates assessed prior to or at the start of pregnancy are presented in eFigures 2 and 3 in Supplement 1.

### Supplemental Analyses

Among 6327 infants exposed to only 1 tetracycline substance, 4952 were exposed to doxycycline only, 1186 were exposed to lymecycline only, and 189 were exposed to tetracycline or oxytetracycline only. The characteristics of infants by tetracycline substance were largely similar to those of infants exposed to any tetracyclines (eTables 5A-5C in Supplement 1). The distribution of covariates was balanced in each subcohort following propensity score matching (eFigures 1B-1D in Supplement 1). The RRs for any MCMs, compared with unexposed infants, were 1.07 (95% CI, 0.93-1.23) for doxycycline only, 0.83 (95% CI, 0.60-1.15) for lymecycline only, and 0.78 (95% CI, 0.32-1.92) for tetracycline or oxytetracycline only (Table 2).

**Table 2.** Association Between First Trimester Tetracycline Exposure and Any Major Congenital Malformations by Tetracycline Substance and Duration of Use

Exposure group	Infants, No.	Cases, No.	Cases/1000 infants (95% CI)	RR (95% CI)
Tetracycline substance				
Unexposed	49 438	1958	39.61 (37.91-41.37)	1 [Reference]
Doxycycline only	4951	209	42.21 (36.86-48.29)	1.07 (0.93-1.23)
Unexposed	11 817	457	38.67 (35.30-42.35)	1 [Reference]
Lymecycline only	1184	38	32.09 (23.11-44.23)	0.83 (0.60-1.15)
Unexposed	1890	64	33.86 (26.37-43.30)	1 [Reference]
Tetracycline or oxytetracycline only	189	5	26.46 (9.78-63.99)	0.78 (0.32-1.92)
Duration of use				
Unexposed	47 138	1792	38.02 (36.32-39.79)	1 [Reference]
Short-term use^a^	4720	198	41.95 (36.49-48.16)	1.10 (0.96-1.27)
Unexposed	13 280	481	36.22 (33.14-39.57)	1 [Reference]
Long-term use^b^	1329	48	36.12 (27.02-47.98)	1.00 (0.75-1.33)

Abbreviation: RR, relative risk.

^a^ Short-term use is defined as less than 30 daily defined doses (DDDs) for doxycycline, less than 15 DDDs for lymecycline, and less than 15 DDDs for tetracycline and oxytetracycline.

^b^ Long-term use is defined as 30 or more DDDs for doxycycline, 15 or more DDDs for lymecycline, and 15 or more DDDs for tetracycline and oxytetracycline.

Of 6252 infants whose mothers filled only 1 prescription for oral formulations of tetracyclines, 4720 infants exposed to short-term tetracycline use and 1329 exposed to long-term use were included in the propensity score–matched cohorts (eTables 5D-5E and eFigures 1E-1F in Supplement 1). Short-term tetracycline users had comorbidities, utilized health care, and used drugs in higher proportions than long-term users. Although 4616 individuals (97.8%) exposed to short-term use were exposed to doxycycline, of those exposed to long-term use, 1029 (77.4%) were exposed to lymecycline, 147 (11.1%) were exposed to tetracycline, 141 (10.6%) were exposed to doxycycline, and 12 (0.9%) were exposed to oxytetracycline. Neither short-term (RR, 1.10; 95% CI, 0.96-1.27) nor long-term (RR, 1.00; 95% CI, 0.75-1.33) use of tetracyclines during the first trimester was associated with increased risks of any MCMs (Table 2).

## Discussion

In this large population-based cohort study comprising more than 6300 tetracycline-exposed, live-born, singleton infants, we investigated the risk of MCMs compared with propensity score–matched unexposed controls. First trimester tetracycline exposure was not associated with increased risks of any MCMs, 10 of 12 MCM subgroups, or any of the 16 investigated individual MCMs. Although higher risks were observed for nervous system anomalies and eye anomalies, these estimates were substantially attenuated in a sensitivity analysis with follow-up extended to age 3 years. On the basis of the upper limits of the 95% CIs, the findings indicate risk increases of no more than 16% for any MCM and no more than 50% for several MCM subgroups, including congenital heart defects, orofacial clefts, malformations of the urinary tract and kidney, genital anomalies, and limb anomalies. Although this study counters prior safety signals and provides new evidence on previously unreported MCM categories, statistical precision remained limited for several MCM subgroups and individual MCMs.

Our neutral finding regarding the risk of any MCM with first trimester exposure to any tetracycline was consistent with that from the only directly comparable population-based cohort study from Quebec, Canada.^15^ While the overall prevalence of any MCM for tetracycline-exposed infants was substantially higher in Quebec (10.5%), the measure of association was similar albeit less precise (odds ratio [OR], 1.04; 95 CI, 0.75-1.43). Notably, their analysis was based on a smaller sample exposed to doxycycline or minocycline (410 individuals), whereas the present study included exposures to primarily doxycycline or lymecycline, and to tetracycline and oxytetracycline to a limited extent. Most other studies investigated doxycycline exposure, detecting no increased risks of any MCMs with first trimester tetracycline exposure across cohorts in the US (1694 exposed; RR, 0.85; 95% CI, 0.59-1.23)^14^ and Denmark (1101 exposed; OR, 1.06; 95% CI, 0.76-1.47),^5^ which our findings confirmed with improved precision. The Canadian study demonstrated an increased risk of any MCMs, although the confidence interval crossed 1 (OR, 1.46; 95% CI, 0.93-2.28).^15^ In addition, although we are not aware of prior studies investigating lymecycline, the risk of any MCMs with this substance was not increased in our study with high precision. Furthermore, we found no evidence of differential risks of any MCMs between short-term and long-term use of tetracyclines during the first trimester.

The present study systematically investigated all MCM subgroups, as classified by EUROCAT, as well as individual malformations of previous concern and those deemed meaningful to analyze given statistical power. These analyses are not directly comparable to prior studies, which focused on some selected tetracycline substances and malformations. We found no increased risk of 10 of the 12 MCM subgroups, and none of the 16 individual malformations studied was associated with first trimester tetracycline exposure. In the main analysis, the risks of nervous system and eye anomalies were 1.9-fold and 1.8-fold higher among exposed infants, respectively, which are previously unreported findings. These did not, however, hold in analyses with a 3-year follow-up period, which is more sensitive for detecting these MCM subgroups.^29^ Thus, the increased risks observed at 1-year follow-up may be explained by differential clinical case detection of these malformations between the exposure groups, rather than true associations. The risk of eye anomalies was reported to attenuate over a 4-year follow-up period in a Nordic study investigating the association between pregabalin exposure and malformations.^30^

For some MCM subgroups and individual MCMs, our findings corroborate those from studies conducted across Canada, Denmark, and the US, confirming null associations for nervous system anomalies,^15^ respiratory anomalies,^15^ congenital heart defects,^5,15,17^ oral clefts,^14,17^ genital anomalies, and craniosynostosis.^15^ Conversely, potential signals previously reported for neural tube defects, cleft lip with or without cleft palate, cleft palate, esophageal atresia or stenosis, ventricular and/or atrial septal defects, and limb reduction defects were not supported by our data, albeit with varying degrees of statistical precision.^7,10,11,12,13,15,16^ We also examined a range of MCMs not previously studied, including ear, neck, and face anomalies; gastrointestinal anomalies; abdominal wall defects; congenital anomalies of kidney and urinary tract; as well as pulmonary valve stenosis, isolated patent ductus arteriosus in full-term infants, congenital hydronephrosis, hypospadias, clubfoot, and polydactyly, observing no new safety concerns.

The present study is substantially larger than previous reports in terms of the number of exposed infants and cases. Coupled with extensive confounding control and sensitivity analyses to test the robustness of our results, our findings expand on previous evidence pertaining to risks of MCMs with improved precision and covering a broad range of MCM subgroups and individual MCMs. Furthermore, we report estimates for tetracycline substances, including lymecycline, which has been underreported in the literature.

### Limitations

Several limitations warrant consideration. Selection bias may have influenced our results as the cohort was sourced from a population of live births; pregnancies that resulted in spontaneous abortion, termination, or stillbirth were not part of the cohort, and MCMs from such pregnancies were, hence, not captured. Our estimates on the occurrence of MCMs may, thus, be underestimated; moreover, the RR estimates may be biased to the null if tetracycline exposure during early pregnancy resulted in MCMs that led to spontaneous abortion or termination of pregnancy.^8,31^ In particular, the prevalence of termination due to prenatally diagnosed neural tube defects is very high (>75% for spina bifida, anencephaly, or similar in European registries),^32^ and the estimates for this outcome from our study should, hence, be interpreted with caution. Our dataset did not include other oral tetracycline substances such as minocycline, or intravenous formulations like tigecycline, underscoring the need for future studies. The absence of data on inpatient antibiotic use, coupled with the lack of information on actual intake of antibiotics or the timing of use, may have led to exposure misclassification. Furthermore, information on indications for which tetracyclines were prescribed was not available, limiting the possibility to account for confounding by indication or to identify suitable active comparators. Such confounding is, nevertheless, unlikely to have biased results toward the null, thereby obscuring a true increased risk, as underlying infections such as sexually transmitted infections for which tetracyclines are prescribed may more likely elevate the risk than protect against selected malformations.^33^ The validity of MCM diagnoses in the Swedish registers is not known. However, in Denmark, a country with a similar health care system and register infrastructure, validation studies^34,35^ report that diagnosis records were correct for 88% of any MCMs and 90% of cardiac defects.

## Conclusions

In this cohort study, first trimester tetracycline exposure was not associated with increased risks of MCMs. Additional studies are still needed to rule out potential risks, owing to power limitations for several MCM subgroups and individual malformations.

## Notes

Supplement 1. **eTable 1. **Study population exclusion criteria **eTable 2.** Definition of major congenital malformations and major malformation groups **eTable 3.** Individual malformations: definition, frequency, prevalence and evaluation of power criteria in the source cohort **eTable 4.** Covariates included in the propensity score estimation **eTable 5.** Characteristics of infants included in the supplementary analyses **eFigure 1.** Standardized mean differences in covariates before and after propensity score matching for the sub cohorts in the main and supplementary analyses **eFigure 2.** Unadjusted analysis on association between first trimester tetracycline exposure and major congenital malformations; based on eligible cohort before propensity score matching **eFigure 3.** Sensitivity analysis on association between first trimester tetracycline exposure and major congenital malformations; with all covariates included in propensity score assessed prior to or at start of pregnancy, but not during first trimester

Supplement 2.
Data Sharing Statement
