# Supplementary material for: First Trimester Tetracycline Exposure and Risk of Major Congenital Malformations
Source: JAMA Netw Open. 2024 Nov 14;7(11):e2445055. doi: 10.1001/jamanetworkopen.2024.45055 (PMC11565264; doi:10.1001/jamanetworkopen.2024.45055)
Supplement: Supplement 1. — eTable 1. Study population exclusion criteria eTable 2. Definition of major congenital malformations and major malformation groups eTable 3. Individual malformations: definition, frequency, prevalence and evaluation of power criteria in the source cohort eTable 4. Covariates included in the propensity score estimation eTable 5. Characteristics of infants included in the supplementary analyses eFigure 1. Standardized mean differences in covariates before and after propensity score matching for the sub cohorts in the main and supplementary analyses eFigure 2. Unadjusted analysis on association between first trimester tetracycline exposure and major congenital malformations; based on eligible cohort before propensity score matching eFigure 3. Sensitivity analysis on association between first trimester tetracycline exposure and major congenital malformations; with all covariates included in propensity score assessed prior to or at start of pregnancy, but not during first trimester [file jamanetwopen-e2445055-s001.pdf]

Supplemental Online Content

Nakitanda AO, Odsbu I, Cesta CE, Pazzagli L, Pasternak B. First trimester tetracycline exposure and risk of major congenital malformations. *JAMA Netw Open*. 2024;7(11):e2445055. doi:10.1001/jamanetworkopen.2024.45055

eTable 1. Study population exclusion criteria

eTable 2. Definition of major congenital malformations and major malformation groups

eTable 3. Individual malformations: definition, frequency, prevalence and evaluation of power criteria in the source cohort

eTable 4. Covariates included in the propensity score estimation

eTable 5. Characteristics of infants included in the supplementary analyses

eFigure 1. Standardized mean differences in covariates before and after propensity score matching for the sub cohorts in the main and supplementary analyses

eFigure 2. Unadjusted analysis on association between first trimester tetracycline exposure and major congenital malformations; based on eligible cohort before propensity score matching

eFigure 3. Sensitivity analysis on association between first trimester tetracycline exposure and major congenital malformations; with all covariates included in propensity score assessed prior to or at start of pregnancy, but not during first trimester

This supplemental material has been provided by the authors to give readers additional information about their work.

© 2024 Nakitanda AO et al. *JAMA Network Open.*

eTable 1. Study population exclusion criteria

	Assessment window	Definition	Source
**Perinatal characteristics**			
Stillborn		Birth record	Medical Birth Register
Multipleton		Antental/birth record	Medical Birth Register
Missing/implausible gestational age at birth		Birth record	Medical Birth Register
**Demographics**			
Non-continuous/Interrupted maternal residence in Sweden	LMP-365 to birth	Immigration and emigration records	Total Population Register
Non-continuous/Interrupted residence in Sweden (Infant)	Birthday to birthday+364	Emigration records	Total Population Register
**Exposure to known teratogens**	LMP-90 to LMP+97	ATC codes	Prescribed Drug Register
Warfarin		B01AA03	
ACEI inhibitors		C09A, C09B	
Angiotensin II reception blockers (ARBs)		C09C, C09D	
Other agents acting on RA system		C09X	
Isotretinoin		D10AD04, D10BA01, D10AD54	
Acitretin		D05BB02	
Alitretinoin		D11AH04	
Other aminoglycosides		J01GB	
Combinations of sulfonamides and trimethoprim		J01EE	
Antineoplastic drugs		L01	
Mycophenolic acid		L04AA06	
Leflunomide		L04AA13	
Teriflunomide		L04AA31	
Thalidomide		L04AX02	
Methotrexate		L04X03, L01BA01	
Lenalidomide		L04AX04	
Pomalidomide		L04AX06	
Phenytoin		N03AB02	
Fosphenytoin		N03AB05	
Ethosuximide		N03AD01	
Clonazepam		N03AE01	
Carbamazepine		N03AF01	
Oxcarbazepine		N03AF02	
Valproic acid		N03AG01	
Vigabatrin		N03AG04	
Topiramate		N03AX11	
Cenobamate		N03AX25	
Lithium		N05AN01, D11AX04	
**Diagnosis of MCM from known causes**	Birthday to birthday+364	ICD-10 codes	National Patient Register, Cause of Death Register
Congenital malformation syndromes due to known exogenous causes, not elsewhere classified		Q86, (Q8680)	
Maternal infections resulting in major malformations		P35.0, P35.1, P35.4, P35.8, P37.1	
Genetic disorders		D82.1, (Q4471), (Q6190), (Q7402), (Q7484), Q75.1, Q75.4, (Q7581), Q77, Q78.0- Q78.9, Q79.6, Q80.0-Q82.4, Q82.8M, (Q8283), Q85.0, Q85.1, Q85.8B, Q87, (Q8934), Q90-Q93, Q96-Q99	

ICD-10 codes in parentheses are codes from the British Paediatric Association without an equivalent code in the standard or Swedish ICD-10.

eTable 2. Definition of major congenital malformations and major malformation groups

Malformation groups included in the definition of the major congenital malformations	ICD-10/ICD-10 SE codes	ICD-10/ICD-10 SE codes of minor anomalies excluded from definition
Nervous system anomalies	Q00-Q07	(Q0461, Q0780, Q0782)
Eye anomalies	Q10-Q15	Q10.1-Q10.3, Q10.5, Q13.5
Ear, face and neck anomalies	Q16-Q18	Q17.0-Q17.5, Q17.9, Q18.0-Q18.2, Q18.4-Q18.7, (Q1880), Q18.9
Congenital heart defects	Q20-Q26	(Q2111), Q24.6, Q25.0 if preterm, (Q2541), Q25.6 if preterm, Q26.1
Respiratory anomalies	Q30, Q32-Q34	Q32.0, Q32.2, (Q33.00), Q33.1
Oro-facial clefts	Q35-Q37	Q35.7
Gastro-intestinal anomalies	Q38-Q45, Q79.0	Q38.1, Q38.2, (Q38.50), Q40.0, Q40.1, (Q4021), Q43.0, (Q4320, Q4381, Q4382), Q44.4, (Q4583)
Abdominal wall defects	Q79.2, Q79.3, Q79.5	
Congenital anomalies of kidney and urinary tract	Q60-Q64, Q79.4	Q61.0, Q62.7, Q63.3
Genital anomalies	Q50-Q56	Q50.1, Q50.2, Q50.5, Q52.3, Q52.5, Q52.7, Q53, Q54.4, (Q5520, Q5521)
Limb anomalies	Q65-Q74	Q65.3-Q65.6, Q66.1-Q66.9, Q67.0-Q67.8, Q68.0, (Q6810, Q6821) Q68.3, Q68.4, Q68.5, (Q7400), Q65.8, Q65.9
Other anomalies /syndromes	Q75.0, (Q7980), Q89.3, (Q8980), Q04.4, (Q0435), Q41.1, Q41.2, Q41.8, Q71.0, Q71.2, (Q7180), Q72.0, Q72.2, (Q7280), Q73.0, Q79.3, (Q7980), (Q7982), Q20.6, Q24.0, (Q3381), Q89.0, Q89.3	

ICD-10 codes in parentheses are codes from the British Paediatric Association without an equivalent code in the standard or Swedish ICD-10.

eTable 3. Individual malformations: definition, frequency, prevalence and evaluation of power criteria in the source cohort

ICD-10 codes	Individual malformations	Frequency, N	Prevalence, N per 1000	Criteria, prevalence≥0.914
Q00, Q01, Q05	Neural tube defects	258	0.21	No
Q00	Anencephaly and similar	8	0.01	No
Q01	Encephalocele and meningocele	42	0.03	No
Q05	Spina Bifida	212	0.17	No
Q03	Hydrocephaly	231	0.19	No
Q02	Severe microcephaly	257	0.21	No
Q04.1, Q04.2	Arhinencephaly /Holoprosencephaly	13	0.01	No
Q04.0	Agenesis of corpus callosum	136	0.11	No
Q11.0, Q11.1, Q11.2	Anophthalmos/Microphthalmos	120	0.1	No
Q11.0, Q11.1	Anophthalmos	18	0.01	No
Q12.0	Congenital cataract	504	0.4	No
Q15.0	Congenital glaucoma	78	0.06	No
Q16.0, Q16.1	Anotia and atresia / stenosis / stricture of external auditory canal	221	0.18	No
Q20.0-Q20.6, Q21.2-Q21.4, Q22.0, Q22.4-Q22.6, Q23.0, Q23.2, Q23.4, Q24.2, Q24.4, Q24.5, Q25.1-Q25.3, Q26.2, Q26.3 except (Q2182)	Severe congenital heart defects	2612	2.1	Yes
Q20.0	Common arterial truncus	67	0.05	No
Q20.1	Double outlet right ventricle	167	0.13	No
Q20.2	Double outlet left ventricle	18	0.01	No
Q20.3	Complete transposition of great arteries (D-TGA)	390	0.31	No
Q20.4	Single ventricle	54	0.04	No
Q20.5	Corrected transposition of great arteries (L-TGA)	51	0.04	No
Q21.0	Ventricular septal defect (VSD)	9900	7.95	Yes
Q21.1 except (Q2111)	Atrial septal defect (ASD)	7733	6.21	Yes
Q21.2	Atrioventricular septal defect (AVSD)	290	0.23	No
Q21.3, Q21.8, (Q2182)	Tetralogy and Pentalogy of Fallot	417	0.33	No
Q22.4	Triscuspid atresia and stenosis	60	0.05	No
Q22.5	Ebstein's anomaly	44	0.04	No
Q22.1	Pulmonary valve stenosis	1308	1.05	Yes
Q22.0	Pulmonary valve atresia	303	0.24	No
Q23.0	Aortic valve atresia/stenosis	406	0.33	No
Q23.2	Mitral valve atresia/stenosis	81	0.07	No
Q23.4	Hypoplastic left heart (HLH/HLHS)	138	0.11	No
Q22.6	Hypoplastic right heart (HRH/HRHS)	52	0.04	No
Q25.1	Coarctation of aorta	769	0.62	No
Q25.2	Aortic atresia /interrupted aortic arch	16	0.01	No
Q26.2	Total anomalous pulmonary venous return	69	0.06	No
Q25.0	Patent Ductus arteriosus (PDA) as only CHD in term infants (GA +37weeks)	1188	0.95	Yes
Q30.0	Choanal stenosis or atresia	127	0.1	No
Q36, Q37	Cleft lip with or without cleft palate	1297	1.04	Yes
Q35 Except Q35.7	Cleft palate	667	0.54	No
Q39.0-Q39.1	Oesophageal atresia with or without tracheo-oesophageal fistula	293	0.24	No
Q41.0	Duodenal atresia or stenosis	167	0.13	No
Q41.1-Q41.8	Atresia or stenosis of other parts of small intestine	115	0.09	No
Q42.0-Q42.3	Ano-rectal atresia or stenosis	432	0.35	No
Q43.1	Hirschsprung's disease	275	0.22	No
Q44.2	Atresia of bile ducts	58	0.05	No
Q45.1	Annular pancreas	21	0.02	No
Q43.3	Anomalies of intestinal fixation	276	0.22	No
Q79.0	Diaphragmatic hernia	262	0.21	No
Q39.0-Q39.1, Q39.3	Oesophageal atresia or stenosis	297	0.24	No
Q79.3	Gastroschisis	216	0.17	No

© 2024 Nakitanda AO et al. *JAMA Network Open.*

ICD-10 codes	Individual malformations	Frequency, N	Prevalence, N per 1000	Criteria, prevalence≥0.914
Q79.2	Omphalocele	96	0.08	No
Q60.0	Unilateral renal agenesis	188	0.15	No
Q60.1, Q60.6	Bilateral renal agenesis including Potter sequence	11	0.01	No
Q60.6	Potter syndrome	13	0.01	No
Q62.0, Q62.1, Q62.3	Congenital hydronephrosis including ureter obstruction	3245	2.6	Yes
Q63.1, Q63.2	Lobulated, fused and horseshoe kidney and ectopic kidney	97	0.08	No
Q64.0, Q64.1	Bladder exstrophy and / or epispadias	62	0.05	No
Q64.2	Posterior urethral valves	215	0.17	No
Q79.4	Prune belly syndrome	2	0	No
Q54 Except Q54.4	Hypospadias (among males)	4105	6.41	Yes
Q56	Indeterminate sex	33	0.03	No
Q71-Q73	Limb reduction defects (LRD)	633	0.51	No
Q71.0, Q71.2, (Q7180), Q72.0, Q72.2, (Q7280), Q73.0	Transverse LRD	79	0.06	No
(Q7131), Q71.4, (Q7231), Q72.5	Longitudinal preaxial LRD	51	0.04	No
Q71.5, Q72.6	Longitudinal postaxial LRD	67	0.05	No
Q71.6, Q72.7	Longitudinal central LRD	46	0.04	No
Q71.1, Q72.1, Q73.1	Intercalary LRD	10	0.01	No
Q66.0	Club foot – talipes equinovarus	1836	1.47	Yes
Q65.0-Q65.2	Hip dislocation	3626	2.91	Yes
Q69	Polydactyly	1419	1.14	Yes
Q70	Syndactyly	979	0.79	No
Q75.0	Craniosynostosis	1318	1.06	Yes
Q89.3	Situs inversus	70	0.06	No
Q04.4	Septo-optic dysplasia	34	0.03	No
(Q0435), Q41.1, Q41.2, Q41.8, Q71.0, Q71.2, (Q7180), Q72.0, Q72,2, (Q7280), Q73.0, Q79.3, (Q7980), (Q7982)	Vascular disruption anomalies	398	0.32	No
Q20.6, Q24.0, (Q3381), Q89.0, Q89.3	Laterality anomalies	156	0.13	No

ICD-10 codes in parentheses are codes from the British Paediatrica Association without an equivalent code in the standard or Swedish ICD-10.

eTable 4. Covariates included in the propensity score estimation

Covariate	Assessment window	Definition	Categories	Source
**Demographics**				
Calendar year of birth		Birth record	2006-2009, 2010-2012, 2013-2015, 2016-2018	Medical Birth Register
Maternal age at birth		Birth record	<20, 20-<25,25-<30, 30-<35, 35-<40, 40-<45, ≥45	Medical Birth Register
Maternal country of birth		Antental record	Sweden, other Nordic country, others, missing	Medical Birth Register
Maternal education in birth year		Highest attained education	Compulsory, Pre-university, Tertiary, missing	LISA
Maternal cohabitation with partner		Antenatal record	Yes/No	Medical Birth Register
**Obstetric history**				
BMI in early pregnancy		Derived from antenatal weight and height measures	<18.5, 18.5-<25, 25-<30, 30-<35, 35-<40, ≥40, missing	Medical Birth Register
Conception through ART		Antenatal record	Yes/No	Medical Birth Register
Any MCM in previous births		As per MCM definition in study	Yes/No	National Patient Register, Cause of death register
**Medical history**				
Pre-exsiting diabetes mellitus	LMP-365 to LMP	Antenatal record and/or ICD-10 codes: E10-14, O24.0, O24.1, O24.2, O24.3	Yes/No	Medical Birth Register, National Patient Register
**Lifestyle**	LMP-365 to LMP+97			
Alcohol use disorder		ICD-10 codes: Z71.4, O35.4, F10	Yes/No	National Patient Register
Other substance use disorder		ICD-10 codes: F11-19, Z71.5, Z86.4	Yes/No	National Patient Register
Smoking during early pregnancy		Antenatal record	Yes/No/missing	Medical Birth Register
**Maternal healthcare utilization**				
Prescription drugs dispensed	LMP-90 to LMP	Number of unique ATC codes (2nd level)	0-1, 2-4, ≥5	Prescribed Drug register
Specialist outpatient visits	LMP-365 to LMP	Number of visits	0, 1-2, ≥3	National Patient register
Hospital admissions	LMP-365 to LMP	Any cause hospital admission	Yes/no	National Patient register
**Maternal drug use**	LMP-90 to LMP+97	ATC codes		
Antihypertensives		C02, C03, C04, C07, C08	Yes/No	Prescribed Drug Register
Antidiabetics		A10	Yes/No	Prescribed Drug Register
Other systemic antimicrobials		J02, J05	Yes/No	Prescribed Drug Register
Antidepressants		N06A	Yes/No	Prescribed Drug Register
Antipsychotics		N05A	Yes/No	Prescribed Drug Register
Benzodiazepines and related drugs		N05BA, N05CD, N05CF	Yes/No	Prescribed Drug Register
Antiepileptics		N03A	Yes/No	Prescribed Drug Register
Opioids		N02A	Yes/No	Prescribed Drug Register

© 2024 Nakitanda AO et al. *JAMA Network Open.*

Covariate	Assessment window	Definition	Categories	Source
NSAIDs		M01A, N02BE01	Yes/No	Prescribed Drug Register
Systemic corticosteroids		H02	Yes/No	Prescribed Drug Register
Oral contraceptives		G03A, G03F	Yes/No	Prescribed Drug Register
Ovulation induction		G03GA, G03GB	Yes/No	Prescribed Drug Register
Folic acid, prescribed use		B03BB01	Yes/No	Prescribed Drug Register
Folic acid, self-reported use		Antenatal record	Yes/No	Medical Birth Register
**Exposure to potential teratogens**	LMP-90 to LMP+97	ATC codes	Yes/No	
Sulfasalazine		A07EC01		Prescribed Drug Register
Colestyramine		C10AC01		Prescribed Drug Register
Adapelene		D10AD03, D10AD53		Prescribed Drug Register
Thiamazole		H03BB02		Prescribed Drug Register
Trimethoprim and derivatives		J01EA01		Prescribed Drug Register
Dimethyl fumarate		L04AX07		Prescribed Drug Register
Diroximel fumarate		L04AX09		Prescribed Drug Register
Antimigraine preparations		N02CC01, N02CC02, N02CC03, N02CC08, N02CD06, N02CX		Prescribed Drug Register
**Bacterial infections in the first trimester**				
Other antibiotic use	LMP to LMP+97	ATC codes: J01 excluding J01XX05 and J01A	Yes/No	Prescribed Drug register
Hospital admission	LMP to LMP+97	ICD-10 codes	Yes/No	National Patient Register
Nervous system		G00-G01, G03 (Except G03.0), G04-G06, A39.0		
Eye and adnexa		H00-H01 (except H01.1), H04.3, H04.4, H05.0, H05.1, H10, A74.0, A36.8, A54.3, B30.3, A39.8, H15, H20, A50.3, A51.4, H44.0, H46		
Ear and mastoid		H60 (except H60.4, H60.5), H62.0, H66, H70		
Circulatory system		I00- I01, I30.1, I31, I33, I38-I39, I40.0, I52.0		
Upper respiratory tract		J00-J06 (Except J02.8, J03.8), J32, J34.0, J35.0, J36, J37, J39.0-J39.9 (Except J39.2, J39.3)		
Pneumonia		J13-J16, J17.0, J18		
Other LRTI including pertussis		A37, J20 (except J20.3-J20.7), J21 (Except J21.0-21.1), J22, J40-42		
Pyothorax/Lung abscess		J85-86		
Digestive system		A00-A05, K20, K35-37, K57, K61, K63.0, K65, K67 (Except 67.3), K75.0, K81, K83.0		
Skin and subcutaneous tissue		L00-L08		
Musculoskeletal and connective tissue		M00, M01 (Except M01.1, M01.4-M01.8), M03, M60.0, M63.0		

© 2024 Nakitanda AO et al. *JAMA Network Open.*

Covariate	Assessment window	Definition	Categories	Source
Genitourinary		N10-12, N29.0, N30 (except N30.4), N34, N39.0, N70-76 (Except N74.1)		
Breast		N61		
STIs		A51-59		
Sepsis		R65.0-R65.1, R65.9		
Other specified bacterial infection		A30-46, A70-74		
Other unspecified bacterial infection		A48, A49		

© 2024 Nakitanda AO et al. *JAMA Network Open.*

eTable 5. Characteristics of infants included in the supplementary analyses

a) Exposure to doxycycline only

Characteristic	Unexposed<br>n= 49 438	Exposed to doxycycline only<br>n= 4 951
**Calendar year of birth**		
2006-2009	16,629 (33.6)	1,638 (33.1)
2010-2012	12,995 (26.3)	1,332 (26.9)
2013-2015	10,880 (22.0)	1,084 (21.9)
2016-2018	8,934 (18.1)	897 (18.1)
**Maternal age at delivery, years**		
<20	1,048 (2.1)	136 (2.7)
20-24	6,684 (13.5)	702 (14.2)
25-29	13,041 (26.4)	1,268 (25.6)
30-34	15,732 (31.8)	1,513 (30.6)
35-39	10,409 (21.1)	1,031 (20.8)
40-44	2,361 (4.8)	280 (5.7)
≥45	163 (0.3)	21 (0.4)
**Maternal country of birth**		
Sweden	39,660 (80.2)	3,982 (80.4)
Other Nordic country	539 (1.1)	44 (0.9)
Others	9,239 (18.7)	925 (18.7)
**Maternal education**		
Compulsory	7,080 (14.3)	761 (15.4)
Pre-university	25,607 (51.8)	2,525 (51.0)
Tertiary	16,341 (33.1)	1,598 (32.3)
Missing	410 (0.8)	67 (1.4)
**Cohabiting partnership**	41,242 (83.4)	4,136 (83.5)
**Body mass index in early pregnancy**		
<18.5, Underweight	1,059 (2.1)	114 (2.3)
18.5-<25.0, Normal	25,287 (51.1)	2,485 (50.2)
25.0-<30.0, Pre-obesity	12,213 (24.7)	1,228 (24.8)
30.0-<35.0, Obesity class I	4,756 (9.6)	499 (10.1)
35.0-<40.0, Obesity class II	1,699 (3.4)	190 (3.8)
≥40.0, Obesity class III	641 (1.3)	76 (1.5)
Missing	3,783 (7.7)	359 (7.3)
**Conception by assisted reproductive therapy**	2,877 (5.8)	292 (5.9)
**Any MCM in previous births (among parous)**	1 184 (4.3)	129 (4.5)
**Other chronic conditions**		
Pre-existing diabetes mellitus	421 (0.9)	43 (0.9)
Alcohol use disorder	268 (0.5)	34 (0.7)
Other substance use disorder	431 (0.9)	55 (1.1)
**Maternal smoking in early pregnancy**		
No	43,351 (87.7)	4,220 (85.2)
Yes	4,330 (8.8)	546 (11.0)
Missing	1,757 (3.6)	185 (3.7)
**Recent healthcare utilization**		
Prescription fills in past 3 months, N (Unique ATC level 2)		
0-1	33,625 (68.0)	3,366 (68.0)
2-4	13,369 (27.0)	1,329 (26.8)
>5	2,444 (4.9)	256 (5.2)
Specialist outpatient visits in past year, N		
0	15,558 (31.5)	1,621 (32.7)
1-2	13,280 (26.9)	1,300 (26.3)
>2	20,600 (41.7)	2,030 (41.0)
Hospitalizations in past year	6,782 (13.7)	698 (14.1)
**Other prescription drug use in past 3 months**		
Antihypertensives	756 (1.5)	76 (1.5)
Antidiabetics	354 (0.7)	36 (0.7)
Antimycotics	1,754 (3.5)	188 (3.8)
Antivirals	1,035 (2.1)	107 (2.2)
Antidepressants	4,044 (8.2)	417 (8.4)

© 2024 Nakitanda AO et al. *JAMA Network Open.*

Characteristic	Unexposed<br>n= 49 438	Exposed to doxycycline only<br>n= 4 951
Antipsychotics	359 (0.7)	43 (0.9)
Benzodiazepines	2,007 (4.1)	221 (4.5)
Antiepileptics	482 (1.0)	54 (1.1)
Opioids	3,270 (6.6)	340 (6.9)
Non-steroidal anti-inflammatory drugs	6,926 (14.0)	706 (14.3)
Systemic corticosteroids	2,656 (5.4)	296 (6.0)
Oral contraceptives	3,140 (6.4)	333 (6.7)
Ovulation induction	3,178 (6.4)	316 (6.4)
Potentially teratogenic drugs	783 (1.6)	87 (1.8)
Prescription folate	1,887 (3.8)	183 (3.7)
Self-reported folate use	8,847 (17.9)	909 (18.4)
**Other antibiotic use during 1st trimester**	11,302 (22.9)	1,101 (22.2)
**Hospital admission for bacterial infection during 1st trimester**	471 (1.0)	70 (1.4)

© 2024 Nakitanda AO et al. *JAMA Network Open.*

b) Exposure to lymecycline only

Characteristic	Unexposed<br>n= 11 817	Exposed to lymecycline only<br>n= 1 184
**Calendar year of birth**		
2006-2009	3,604 (30.5)	346 (29.2)
2010-2012	2,948 (24.9)	296 (25.0)
2013-2015	2,668 (22.6)	305 (25.8)
2016-2018	2,597 (22.0)	237 (20.0)
**Maternal age at delivery, years**		
<20	264 (2.2)	19 (1.6)
20-24	1,697 (14.4)	177 (14.9)
25-29	3,678 (31.1)	375 (31.7)
30-34	3,926 (33.2)	391 (33.0)
35-39	1,852 (15.7)	182 (15.4)
40-44	386 (3.3)	39 (3.3)
≥45	14 (0.1)	1 (0.1)
**Maternal country of birth**		
Sweden	9,751 (82.5)	972 (82.1)
Other Nordic country	87 (0.7)	6 (0.5)
Others	1,979 (16.7)	206 (17.4)
**Maternal education**		
Compulsory	1,510 (12.8)	147 (12.4)
Pre-university	5,919 (50.1)	613 (51.8)
Tertiary	4,309 (36.5)	418 (35.3)
Missing	79 (0.7)	6 (0.5)
**Cohabiting partnership**	10,047 (85.0)	1,005 (84.9)
**Body mass index in early pregnancy**		
<18.5, Underweight	262 (2.2)	32 (2.7)
18.5-<25.0, Normal	7,191 (60.9)	743 (62.8)
25.0-<30.0, Pre-obesity	2,417 (20.5)	213 (18.0)
30.0-<35.0, Obesity class I	796 (6.7)	68 (5.7)
35.0-<40.0, Obesity class II	246 (2.1)	24 (2.0)
≥40.0, Obesity class III	116 (1.0)	13 (1.1)
Missing	789 (6.7)	91 (7.7)
**Conception by assisted reproductive therapy**	316 (2.7)	31 (2.6)
**Any MCM in previous births (among parous)**	128 (2.1)	14 (2.8)
**Other chronic conditions**		
Pre-existing diabetes mellitus	72 (0.6)	7 (0.6)
Alcohol use disorder	98 (0.8)	11 (0.9)
Other substance use disorder	68 (0.6)	8 (0.7)
**Maternal smoking in early pregnancy**		
No	10,628 (89.9)	1,072 (90.5)
Yes	712 (6.0)	65 (5.5)
Missing	477 (4.0)	47 (4.0)
**Recent healthcare utilization**		
Prescription fills in past 3 months, N (Unique ATC 2nd level)		
0-1	7,693 (64.9)	756 (63.7)
2-4	3,295 (27.8)	339 (28.6)
>5	863 (7.3)	91 (7.7)
Specialist outpatient visits in past year, N		
0	3,224 (27.2)	314 (26.5)
1-2	3,692 (31.2)	393 (33.1)
>2	4,935 (41.6)	479 (40.4)
Hospitalizations in past year	1,446 (12.2)	132 (11.1)
**Other prescription drug use in past year**		
Antihypertensives	152 (1.3)	15 (1.3)
Antidiabetics	89 (0.8)	9 (0.8)

© 2024 Nakitanda AO et al. *JAMA Network Open.*

Antimycotics	623 (5.3)	64 (5.4)
Antivirals	300 (2.5)	31 (2.6)
Antidepressants	1,284 (10.9)	134 (11.3)
Antipsychotics	110 (0.9)	11 (0.9)
Benzodiazepines	476 (4.0)	52 (4.4)
Antiepileptics	134 (1.1)	16 (1.4)
Opioids	542 (4.6)	56 (4.7)
Non-steroidal anti-inflammatory drugs	1,006 (8.5)	100 (8.4)
Systemic corticosteroids	403 (3.4)	44 (3.7)
Oral contraceptives	632 (5.3)	66 (5.6)
Ovulation induction	343 (2.9)	33 (2.8)
Potentially teratogenic drugs	2,142 (18.1)	216 (18.2)
Prescription folate	193 (1.6)	19 (1.6)
Self-reported folate use	2,061 (17.4)	206 (17.4)
**Other antibiotic use during 1st trimester**	1,358 (11.5)	131 (11.1)
**Hospital admission for bacterial infection during 1st trimester**	11 (0.1)	1 (0.1)

© 2024 Nakitanda AO et al. *JAMA Network Open.*

c) Exposure to tetracycline or oxytetracycline only

Characteristic	Unexposed<br><br>n= 1 890	Exposed to tetracycline only or oxytetracycline only<br>n= 189
**Calendar year of birth**		
2006-2009	756 (40)	73 (38.6)
2010-2012	587 (31.1)	61 (32.3)
2013-2015	328 (17.4)	29 (15.3)
2016-2018	219 (11.6)	26 (13.8)
**Maternal age at delivery, years**		
<20	13 (0.7)	3 (1.6)
20-24	273 (14.4)	27 (14.3)
25-29	615 (32.5)	57 (30.2)
30-34	569 (30.1)	58 (30.7)
35-39	334 (17.7)	32 (16.9)
40-44	83 (4.4)	10 (5.3)
≥45	3 (0.2)	<3 (<1.2)
**Maternal country of birth**		
Sweden	1,506 (79.7)	150 (79.4)
Other Nordic country	52 (2.8)	8 (4.2)
Others	332 (17.6)	31 (16.4)
**Maternal education**		
Compulsory	224 (11.9)	20 (10.6)
Pre-university	993 (52.5)	104 (55.0)
Tertiary	634 (33.5)	58 (30.7)
Missing	39 (2.1)	7 (3.7)
**Cohabiting partnership**	1,640 (86.8)	163 (86.2)
**Body mass index in early pregnancy**		
<18.5, Underweight	42 (2.2)	3 (1.6)
18.5-<25.0, Normal	1,249 (66.1)	127 (67.2)
25.0-<30.0, Pre-obesity	376 (19.9)	35 (18.5)
30.0-<35.0, Obesity class I	117 (6.2)	7 (3.7)
35.0-<40.0, Obesity class II	42 (2.2)	7 (3.7)
≥40.0, Obesity class III	4 (0.2)	<3 (<1.2)
Missing	60 (3.2)	8 (4.2)
**Conception by assisted reproductive technology**	42 (2.2)	4 (2.1)
**Any MCM in previous births (among parous)**	20 (1.9)	3 (3.3)
**Other chronic conditions**		
Pre-existing diabetes mellitus	8 (0.4)	<3 (<1.0)
Alcohol use disorder	0	0
Other substance use disorder	0	0
**Maternal smoking in early pregnancy**		
No	1,680 (88.9)	163 (86.2)
Yes	169 (8.9)	20 (10.6)
Missing	41 (2.2)	6 (3.2)
**Recent healthcare utilization**		
Prescription fills in past 3months, N (Unique ATC 2nd level)		
0-1	1,250 (66.1)	127 (67.2)
2-4	536 (28.4)	53 (28.0)
>5	104 (5.5)	9 (4.8)
Specialist outpatient visits in past year, N		
0	582 (30.8)	59 (31.2)
1-2	646 (34.2)	67 (35.4)
>2	662 (35.0)	63 (33.3)
Hospitalizations in past year	247 (13.1)	22 (11.6)
**Other maternal prescription drug use**		
Antihypertensives	39 (2.1)	4 (2.1)

Characteristic	Unexposed<br><br>n= 1 890	Exposed to tetracycline only or oxytetracycline only<br>n= 189
Antidiabetics	8 (0.4)	<3 (<1.0)
Antimycotics	81 (4.3)	8 (4.2)
Antivirals	37 (2.0)	5 (2.6)
Antidepressants	209 (11.1)	24 (12.7)
Antipsychotics	0	0
Benzodiazepines	129 (6.8)	13 (6.9)
Antiepileptics	34 (1.8)	4 (2.1)
Opioids	99 (5.2)	10 (5.3)
Non-steroidal anti-inflammatory drugs	250 (13.2)	20 (10.6)
Systemic corticosteroids	37 (2.0)	<3 (<1.2)
Oral contraceptives	94 (5.0)	10 (5.3)
Ovulation induction	49 (2.6)	7 (3.7)
Potentially teratogenic drugs	99 (5.2)	10 (5.3)
Prescription folate	76 (4.0)	9 (4.8)
Self-reported folate use	204 (10.8)	22 (11.6)
**Other antibiotic use during 1st trimester**	261 (13.8)	24 (12.7)
**Hospital admission for bacterial infection in 1st trimester**	0	0

© 2024 Nakitanda AO et al. *JAMA Network Open.*

d) Exposure to short-term tetracycline use

Characteristic	Unexposed<br>n= 47 138	Exposed to short-term use<br>n= 4 720
**Calendar year of birth**		
2006-2009	16 622 (35.3)	1 617 (34.3)
2010-2012	12 345 (26.2)	1 265 (26.8)
2013-2015	10 131 (21.5)	1 009 (21.4)
2016-2018	8 040 (17.1)	829 (17.6)
**Maternal age at delivery, years**		
<20	1 094 (2.3)	137 (2.9)
20-24	6 835 (14.5)	693 (14.7)
25-29	12 465 (26.4)	1 204 (25.5)
30-34	14 587 (30.9)	1 427 (30.2)
35-39	9 750 (20.7)	972 (20.6)
40-44	2 264 (4.8)	266 (5.6)
≥45	143 (0.3)	21 (0.4)
**Maternal country of birth**		
Sweden	38 019 (80.7)	3 808 (80.7)
Other Nordic country	488 (1.0)	42 (0.9)
Others	8 631 (18.3)	870 (18.4)
**Maternal education**		
Compulsory	6 893 (14.6)	745 (15.8)
Pre-university	24 634 (52.3)	2 426 (51.4)
Tertiary	15 190 (32.2)	1 480 (31.4)
Missing	421 (0.9)	69 (1.5)
**Cohabiting partnership**	39 249 (83.3)	3 935 (83.4)
**Body mass index in early pregnancy**		
<18.5, Underweight	23 837 (50.6)	2 351 (49.8)
18.5-<25.0, Normal	1 031 (2.2)	107 (2.3)
25.0-<30.0, Pre-obesity	11 535 (24.5)	1 178 (25)
30.0-<35.0, Obesity class I	4 778 (10.1)	481 (10.2)
35.0-<40.0, Obesity class II	1 630 (3.5)	185 (3.9)
≥40.0, Obesity class III	613 (1.3)	72 (1.5)
Missing	3 714 (7.9)	346 (7.3)
**Conception by assisted reproductive therapy**	2 769 (5.9)	278 (5.9)
**Any MCM in previous births (among parous)**	1 104 (4.2)	120 (4.4)
**Other chronic conditions**		
Pre-existing diabetes mellitus	407 (0.9)	42 (0.9)
Alcohol use disorder	290 (0.6)	33 (0.7)
Other substance use disorder	432 (0.9)	53 (1.1)
**Maternal smoking in early pregnancy**		
No	41 116 (87.2)	3 996 (84.7)
Yes	4 203 (8.9)	543 (11.5)
Missing	1 819 (3.9)	181 (3.8)
**Recent healthcare utilization**		
Prescription fills in past 3 months, N (Unique ATC 2nd level)		
0-1	32 076 (68.0)	3 215 (68.1)
2-4	12 740 (27.0)	1 268 (26.9)
>5	2 322 (4.9)	237 (5)
Specialist outpatient visits in past year, N		
0	14 842 (31.5)	1 538 (32.6)
1-2	12 643 (26.8)	1 247 (26.4)
>2	19 653 (41.7)	1 935 (41.0)
Hospitalizations in past year	6 559 (13.9)	666 (14.1)
**Other prescription drug use in past year**		
Antihypertensives	691 (1.5)	69 (1.5)

Characteristic	Unexposed<br><br>n= 47 138	Exposed to short-term use<br>n= 4 720
Antidiabetics	348 (0.7)	35 (0.7)
Antimycotics	1 722 (3.7)	191 (4.0)
Antivirals	926 (2.0)	96 (2.0)
Antidepressants	3 988 (8.5)	402 (8.5)
Antipsychotics	347 (0.7)	42 (0.9)
Benzodiazepines	1 892 (4.0)	206 (4.4)
Antiepileptics	441 (0.9)	51 (1.1)
Opioids	2 921 (6.2)	321 (6.8)
Non-steroidal anti-inflammatory drugs	6 547 (13.9)	672 (14.2)
Systemic corticosteroids	2 567 (5.4)	283 (6.0)
Oral contraceptives	3 215 (6.8)	321 (6.8)
Ovulation induction	2 988 (6.3)	304 (6.4)
Potentially teratogenic drugs	768 (1.6)	82 (1.7)
Prescription folate	1 626 (3.4)	169 (3.6)
Self-reported folate use	8 324 (17.7)	861 (18.2)
**Other antibiotic use during 1st trimester**	10 828 (23.0)	1 063 (22.5)
**Hospital admission for bacterial infection during 1st trimester**	448 (1.0)	65 (1.4)

© 2024 Nakitanda AO et al. *JAMA Network Open.*

e) Exposure to long-term tetracycline use

Characteristic	Unexposed<br><br>n= 13 280	Exposed to long-term use<br>n= 1 329
**Calendar year of birth**		
2006-2009	3 981 (30.0)	370 (27.8)
2010-2012	3 349 (25.2)	347 (26.1)
2013-2015	2 953 (22.2)	332 (25.0)
2016-2018	2 997 (22.6)	280 (21.1)
**Maternal age at delivery, years**		
<20	184 (1.4)	18 (1.4)
20-24	1 753 (13.2)	183 (13.8)
25-29	4 224 (31.8)	425 (32.0)
30-34	4 441 (33.4)	441 (33.2)
35-39	2 225 (16.8)	212 (16.0)
40-44	434 (3.3)	47 (3.5)
≥45	19 (0.1)	3 (0.2)
**Maternal country of birth**		
Sweden	10 728 (80.8)	1 072 (80.7)
Other Nordic country	144 (1.1)	10 (0.8)
Others	2 408 (18.1)	247 (18.6)
**Maternal education**		
Compulsory	1 620 (12.2)	155 (11.7)
Pre-university	6 533 (49.2)	678 (51.0)
Tertiary	5 031 (37.9)	487 (36.6)
Missing	96 (0.7)	9 (0.7)
**Cohabiting partnership**	11 365 (85.6)	1 136 (85.5)
**Body mass index in early pregnancy**		
<18.5, Underweight	8 214 (61.9)	847 (63.7)
18.5-<25.0, Normal	305 (2.3)	37 (2.8)
25.0-<30.0, Pre-obesity	2 681 (20.2)	235 (17.7)
30.0-<35.0, Obesity class I	849 (6.4)	71 (5.3)
35.0-<40.0, Obesity class II	300 (2.3)	30 (2.3)
≥40.0, Obesity class III	142 (1.1)	15 (1.1)
Missing	789 (5.9)	94 (7.1)
**Conception by assisted reproductive technology**	353 (2.7)	37 (2.8)
**Any MCM in previous births (among parous)**	188 (2.7)	20 (3.5)
**Other chronic conditions**		
Pre-existing diabetes mellitus	74 (0.6)	7 (0.5)
Alcohol use disorder	87 (0.7)	10 (0.8)
Other substance use disorder	63 (0.5)	8 (0.6)
**Maternal smoking in early pregnancy**		
No	11 981 (90.2)	1 210 (91)
Yes	836 (6.3)	73 (5.5)
Missing	463 (3.5)	46 (3.5)
**Recent healthcare utilization**		
Prescription fills in past 3months, N (Unique ATC 2nd level)		
0-1	9 052 (68.2)	897 (67.5)
2-4	3 655 (27.5)	366 (27.5)
>5	573 (4.3)	66 (5.0)
Specialist outpatient visits in past year, N		
0	3 694 (27.8)	389 (29.3)
1-2	4 284 (32.3)	438 (33.0)
>2	5 302 (39.9)	502 (37.8)
Hospitalizations in past year	1 478 (11.1)	149 (11.2)
**Other maternal prescription drug use**		
Antihypertensives	165 (1.2)	16 (1.2)

Characteristic	Unexposed<br><br>n= 13 280	Exposed to long-term use<br>n= 1 329
Antidiabetics	96 (0.7)	10 (0.8)
Antimycotics	518 (3.9)	56 (4.2)
Antivirals	379 (2.9)	37 (2.8)
Antidepressants	1 320 (9.9)	140 (10.5)
Antipsychotics	78 (0.6)	10 (0.8)
Benzodiazepines	626 (4.7)	65 (4.9)
Antiepileptics	149 (1.1)	16 (1.2)
Opioids	517 (3.9)	60 (4.5)
Non-steroidal anti-inflammatory drugs	1 102 (8.3)	114 (8.6)
Systemic corticosteroids	421 (3.2)	46 (3.5)
Oral contraceptives	746 (5.6)	75 (5.6)
Ovulation induction	419 (3.2)	39 (2.9)
Potentially teratogenic drugs	2 120 (16.0)	212 (16.0)
Prescription folate	336 (2.5)	34 (2.6)
Self-reported folate use	2 281 (17.2)	227 (17.1)
**Other antibiotic use during 1st trimester**	1 585 (11.9)	142 (10.7)
**Hospital admission for bacterial infection in 1st trimester**	11 (0.1)	<3 (<0.1)

© 2024 Nakitanda AO et al. *JAMA Network Open.*

eFigure 1. Standardized mean differences in covariates before and after propensity score matching for the sub cohorts in the main and supplementary analyses

a. Main analysis: Tetracycline exposed Vs Unexposed

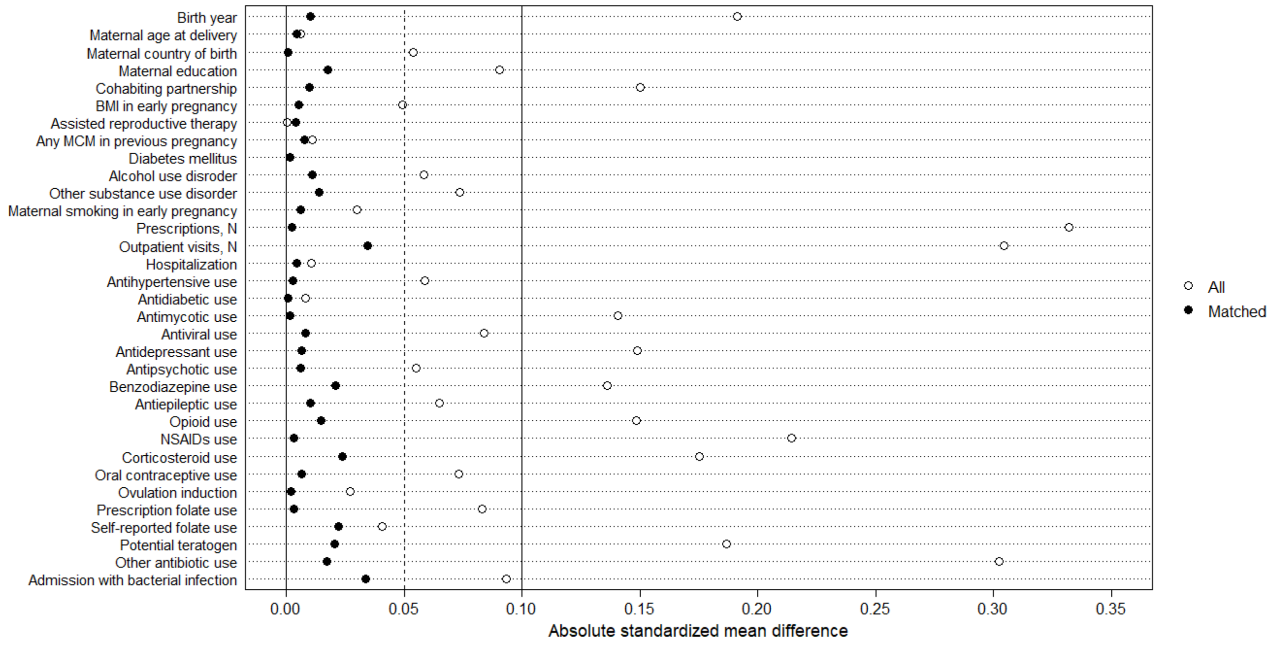

b. Supplementary analysis: Doxycycline only Vs Unexposed

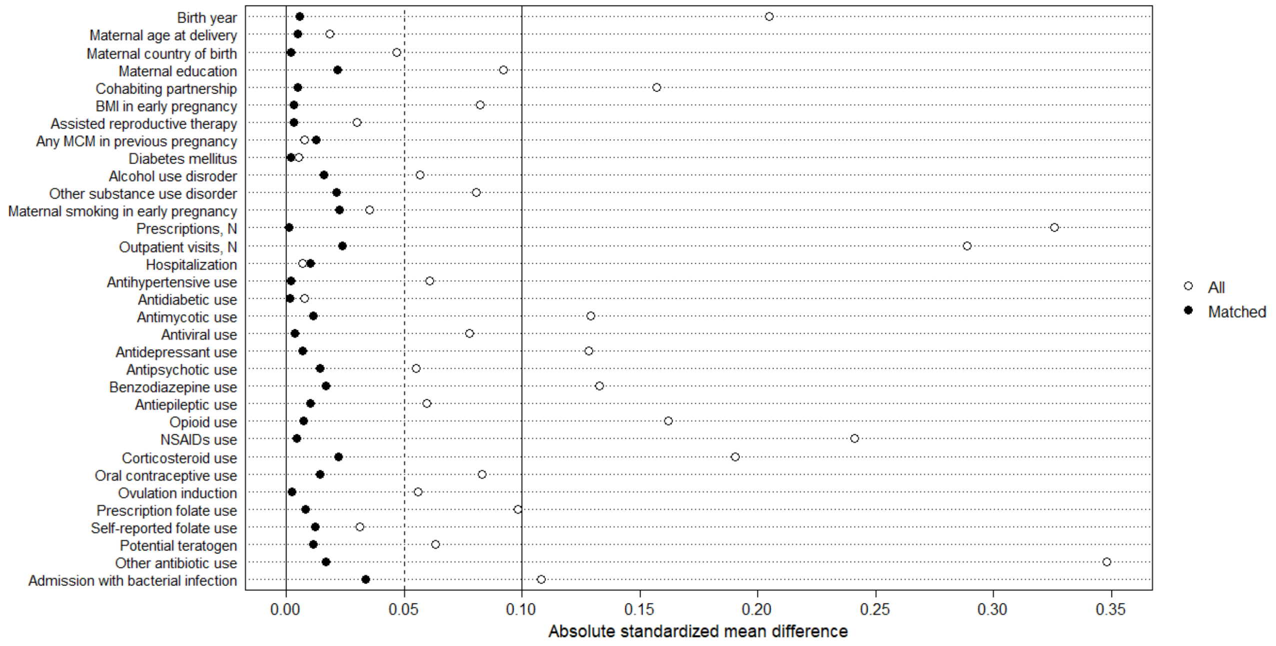

© 2024 Nakitanda AO et al. *JAMA Network Open.*

c. Supplementary analysis: Lymecycline only Vs Unexposed

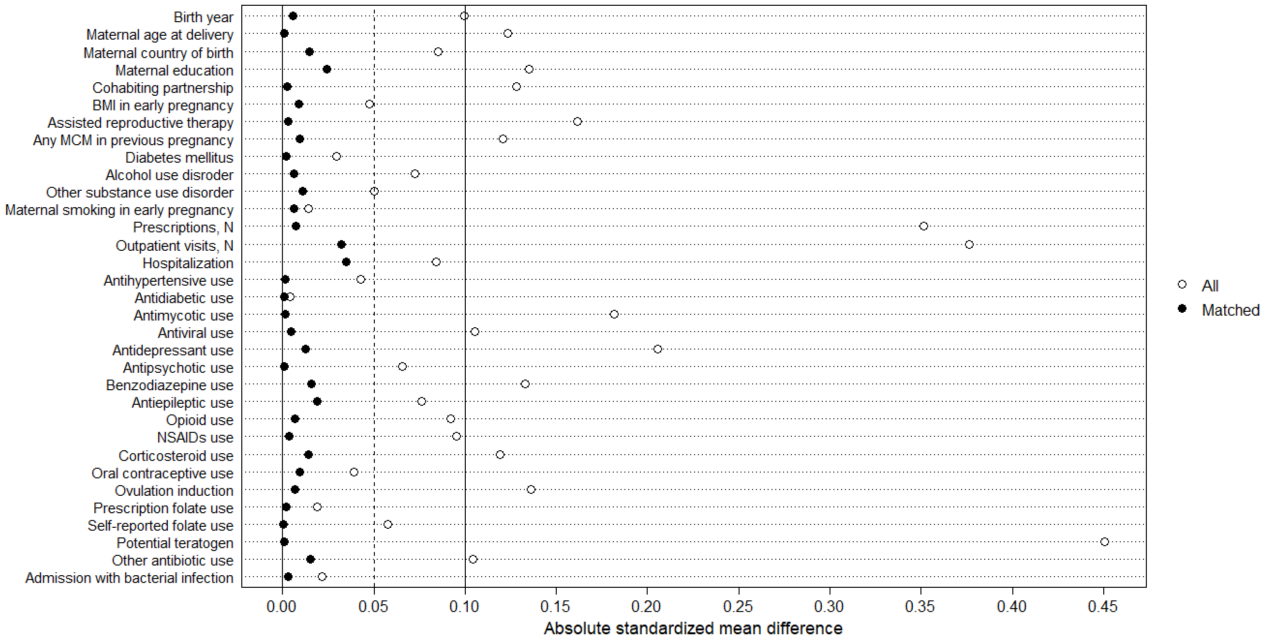

d. Supplementary analysis: Tetracycline or oxytetracycline only Vs Unexposed

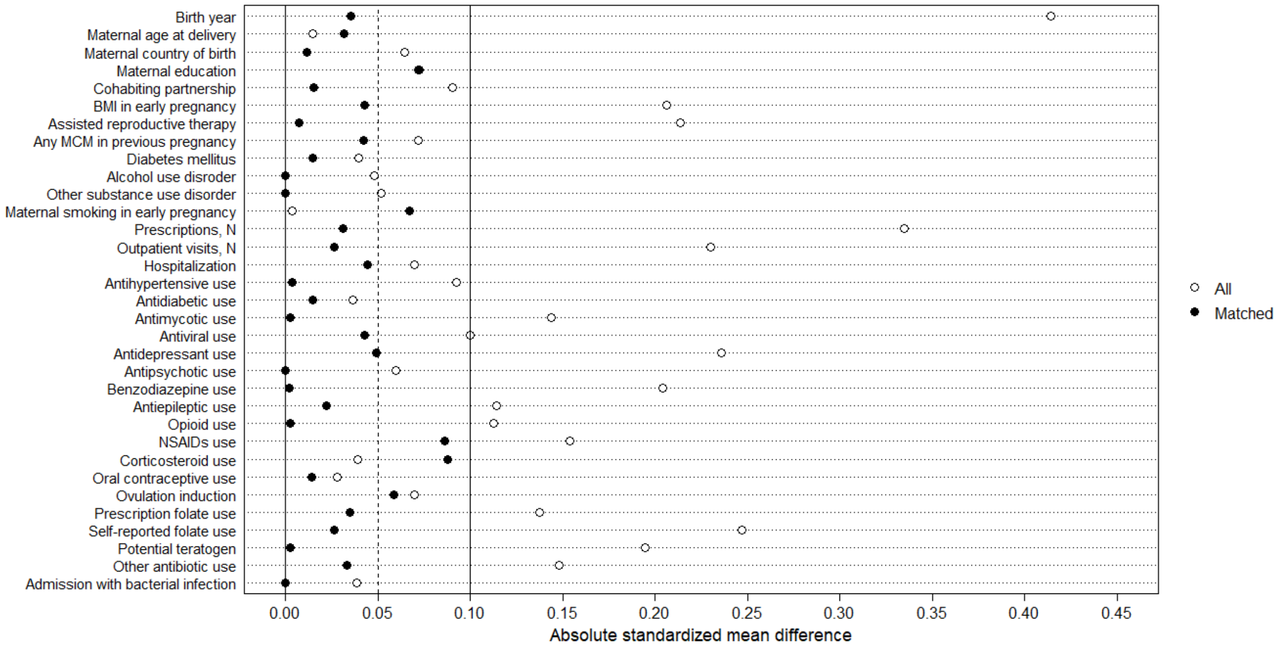

© 2024 Nakitanda AO et al. *JAMA Network Open.*

e. Supplementary analysis: Short-term tetracycline exposure Vs Unexposed

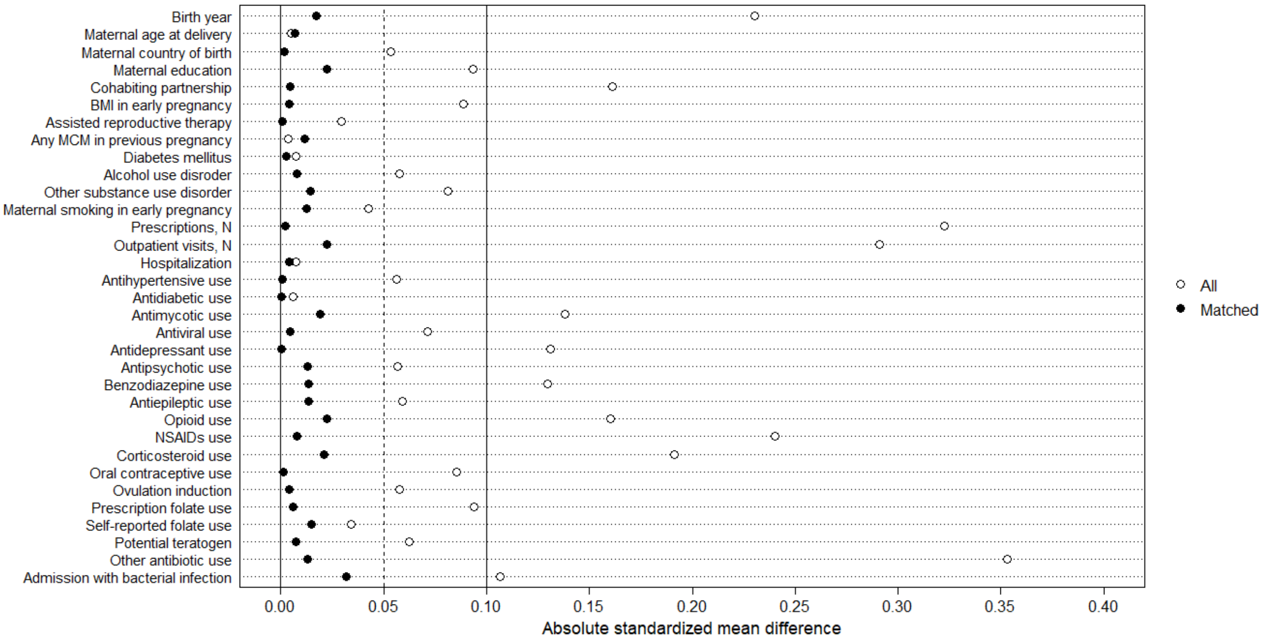

f. Supplementary analysis: Long-term tetracycline exposure Vs Unexposed

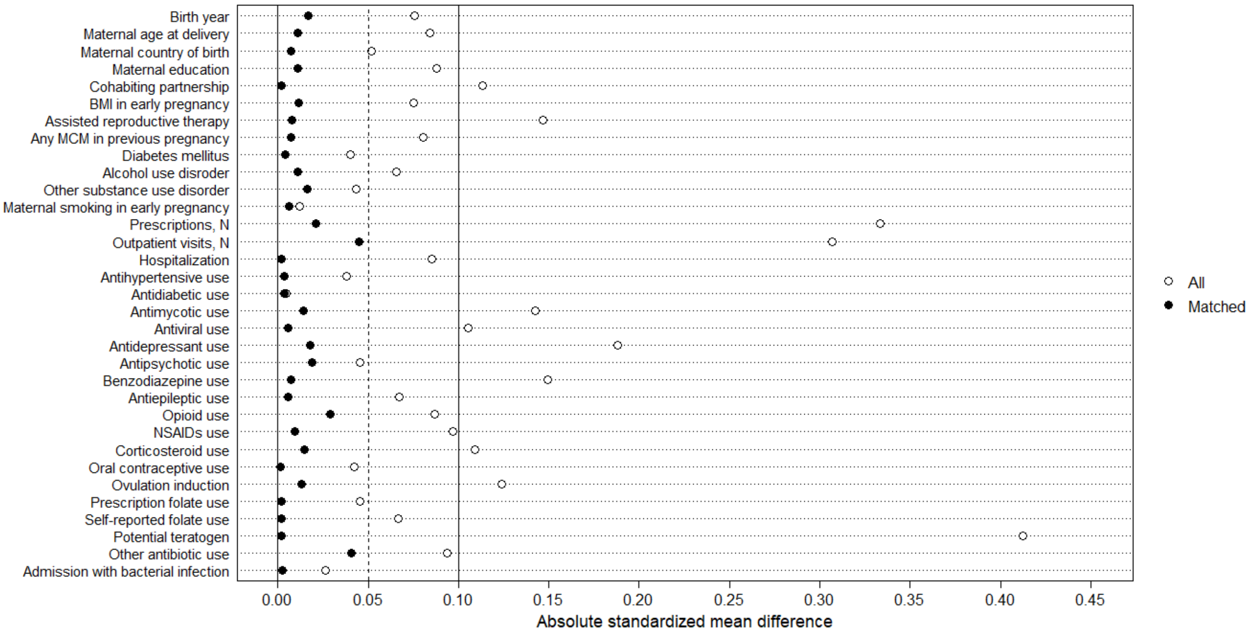

© 2024 Nakitanda AO et al. *JAMA Network Open.*

eFigure 2. Unadjusted analysis on the association between first trimester tetracycline exposure and major congenital malformations; based on eligible cohort before propensity score matching

Malformation	Unexposed, N = 1 239 548 Cases, N	Unexposed, N = 1 239 548 N per 1000 (95% CI)	Exposed, N = 6 341 Cases, N	Exposed, N = 6 341 N per 1000 (95% CI)	Relative Risk (95% CI)
Any major malformation	46623	37.61 (37.28-37.95)	252	39.74 (35.13-44.92)	1.06 (0.94-1.19)
Nervous system anomalies	1254	1.01 (0.96- 1.07)	10	1.58 (0.80- 3.00)	1.56 (0.84-2.90)
Neural tube defects	257	0.21 (0.18- 0.23)	<3	0.16 (0.01- 1.02)	0.76 (0.11-5.42)
Eye anomalies	2451	1.98 (1.90- 2.06)	18	2.84 (1.74- 4.58)	1.44 (0.90-2.28)
Ear, neck and face anomalies	520	0.42 (0.38- 0.46)	<3	0.32 (0.05- 1.27)	0.75 (0.19-3.01)
Congenital heart defects	19062	15.38 (15.16-15.60)	104	16.40 (13.48-19.92)	1.07 (0.88-1.29)
Severe congenital heart defects	2601	2.10 (2.02- 2.18)	11	1.73 (0.91- 3.20)	0.83 (0.46-1.49)
Ventricular septal defect	9853	7.95 (7.79- 8.11)	47	7.41 (5.51- 9.93)	0.93 (0.70-1.24)
Atrial septal defect	7688	6.20 (6.07- 6.34)	45	7.10 (5.24- 9.57)	1.14 (0.85-1.53)
Pulmonary valve stenosis	1300	1.05 (0.99- 1.11)	8	1.26 (0.59- 2.59)	1.20 (0.60-2.41)
Isolated patent ductus arteriosus in term infants	1183	0.95 (0.90- 1.01)	5	0.79 (0.29- 1.95)	0.83 (0.34-1.99)
Respiratory anomalies	500	0.40 (0.37- 0.44)	3	0.47 (0.12- 1.51)	1.17 (0.38-3.65)
Oro-facial clefts	1770	1.43 (1.36- 1.50)	8	1.26 (0.59- 2.59)	0.88 (0.44-1.77)
Cleft lip with or without cleft palate	1289	1.04 (0.98- 1.10)	8	1.26 (0.59- 2.59)	1.21 (0.61-2.43)
Cleft palate	667	0.54 (0.50- 0.58)	0		
Gastrointestinal anomalies	3118	2.52 (2.43- 2.61)	13	2.05 (1.14- 3.60)	0.82 (0.47-1.40)
Oesophageal atresia or stenosis	296	0.24 (0.21- 0.27)	<3	0.16 (0.01- 1.02)	0.66 (0.09-4.70)
Abdominal wall defects	321	0.26 (0.23- 0.29)	<3	0.32 (0.05- 1.27)	1.22 (0.30-4.89)
Congenital anomalies of kidney and urinary tract	4599	3.71 (3.60- 3.82)	21	3.31 (2.10- 5.15)	0.89 (0.58-1.37)
Congenital hydronephrosis including ureter obstruction	3227	2.60 (2.51- 2.70)	18	2.84 (1.74- 4.58)	1.09 (0.69-1.73)
Genital anomalies	5750	4.64 (4.52- 4.76)	29	4.57 (3.12- 6.65)	0.99 (0.69-1.42)
Hypospadias*	4082	6.40 (6.21- 6.60)	23	6.92 (4.49-10.54)	1.08 (0.72-1.63)
Limb anomalies	8667	6.99 (6.85- 7.14)	44	6.94 (5.11- 9.39)	0.99 (0.74-1.33)
Limb reduction defects	628	0.51 (0.47- 0.55)	5	0.79 (0.29- 1.95)	1.56 (0.65-3.75)
Club foot	1824	1.47 (1.41- 1.54)	12	1.89 (1.03- 3.41)	1.29 (0.73-2.27)
Hip dislocation	3614	2.92 (2.82- 3.01)	12	1.89 (1.03- 3.41)	0.65 (0.37-1.14)
Polydactyly	1411	1.14 (1.08- 1.20)	8	1.26 (0.59- 2.59)	1.11 (0.55-2.22)
Other anomalies/syndromes	1889	1.52 (1.46- 1.59)	14	2.21 (1.26- 3.80)	1.45 (0.86-2.45)
Craniosynostosis	1307	1.05 (1.00- 1.11)	11	1.73 (0.91- 3.20)	1.65 (0.91-2.98)

*Male population: Unexposed N = 637 567, Exposed N = 3 325

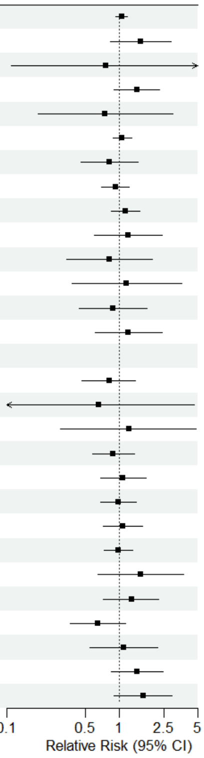

© 2024 Nakitanda AO et al. *JAMA Network Open.*

eFigure 3. Sensitivity analysis on association between first trimester tetracycline exposure and major congenital malformations; with all covariates included in propensity score assessed prior to or at start of pregnancy, but not during first trimester

Malformation	Unexposed, N = 63 350<br>Cases, N	Unexposed, N = 63 350<br>N per 1000 (95% CI)	Exposed, N = 6 339<br>Cases, N	Exposed, N = 6 339<br>N per 1000 (95% CI)	Relative Risk (95% CI)
Any major malformation	2405	37.96 (36.50-39.49)	252	39.75 (35.14-44.93)	1.05 (0.92-1.19)
Nervous system anomalies	55	0.87 (0.66- 1.14)	10	1.58 (0.80- 3.00)	1.82 (0.93-3.56)
Neural tube defects	11	0.17 (0.09- 0.32)	<3	0.16 (0.01- 1.02)	0.91 (0.12-7.04)
Eye anomalies	108	1.70 (1.41- 2.07)	18	2.84 (1.74- 4.58)	1.67 (1.01-2.74)
Ear, neck and face anomalies	28	0.44 (0.30- 0.65)	<3	0.32 (0.05- 1.27)	0.71 (0.17-3.00)
Congenital heart defects	972	15.34 (14.41-16.34)	104	16.41 (13.49-19.93)	1.07 (0.88-1.31)
Severe congenital heart defects	130	2.05 (1.72- 2.44)	11	1.74 (0.91- 3.21)	0.85 (0.46-1.56)
Ventricular septal defect	516	8.15 (7.47- 8.88)	47	7.41 (5.51- 9.93)	0.91 (0.68-1.22)
Atrial septal defect	413	6.52 (5.91- 7.18)	45	7.10 (5.24- 9.58)	1.09 (0.80-1.48)
Pulmonary valve stenosis	69	1.09 (0.85- 1.39)	8	1.26 (0.59- 2.59)	1.16 (0.56-2.41)
Isolated patent ductus arteriosus in term infants	47	0.74 (0.55- 1.00)	5	0.79 (0.29- 1.96)	1.06 (0.42-2.67)
Respiratory anomalies	19	0.30 (0.19- 0.48)	3	0.47 (0.12- 1.51)	1.58 (0.47-5.33)
Oro-facial clefts	105	1.66 (1.36- 2.01)	8	1.26 (0.59- 2.59)	0.76 (0.37-1.56)
Cleft lip with or without cleft palate	78	1.23 (0.98- 1.54)	8	1.26 (0.59- 2.59)	1.02 (0.50-2.12)
Cleft palate	44	0.69 (0.51- 0.94)	0		
Gastrointestinal anomalies	140	2.21 (1.87- 2.62)	13	2.05 (1.14- 3.61)	0.93 (0.53-1.64)
Oesophageal atresia or stenosis	11	0.17 (0.09- 0.32)	<3	0.16 (0.01- 1.02)	0.91 (0.12-7.04)
Abdominal wall defects	21	0.33 (0.21- 0.52)	<3	0.32 (0.05- 1.27)	0.95 (0.22-4.06)
Congenital anomalies of kidney and urinary tract	241	3.80 (3.35- 4.32)	21	3.31 (2.11- 5.15)	0.87 (0.56-1.36)
Congenital hydronephrosis including ureter obstruction	161	2.54 (2.17- 2.97)	18	2.84 (1.74- 4.58)	1.12 (0.69-1.82)
Genital anomalies	298	4.70 (4.19- 5.28)	29	4.57 (3.12- 6.66)	0.97 (0.66-1.42)
Hypospadias*	200	6.03 (5.23- 6.93)	23	6.92 (4.49-10.54)	1.15 (0.75-1.76)
Limb anomalies	455	7.18 (6.55- 7.88)	44	6.94 (5.11- 9.39)	0.97 (0.71-1.32)
Limb reduction defects	38	0.60 (0.43- 0.83)	5	0.79 (0.29- 1.96)	1.31 (0.52-3.34)
Club foot	112	1.77 (1.46- 2.14)	12	1.89 (1.03- 3.41)	1.07 (0.59-1.94)
Hip dislocation	163	2.57 (2.20- 3.01)	12	1.89 (1.03- 3.41)	0.74 (0.41-1.32)
Polydactyly	79	1.25 (0.99- 1.56)	8	1.26 (0.59- 2.59)	1.01 (0.49-2.09)
Other anomalies/syndromes	117	1.85 (1.53- 2.22)	14	2.21 (1.26- 3.80)	1.20 (0.69-2.08)
Craniosynostosis	81	1.28 (1.02- 1.60)	11	1.74 (0.91- 3.21)	1.36 (0.72-2.55)

*Male population: Unexposed N = 33 193, Exposed N = 3 325

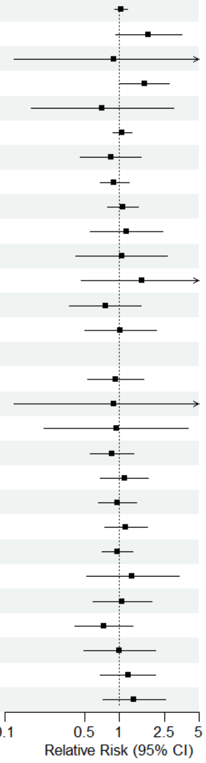

All covariates included in propensity score estimation were assessed prior to or at start of pregnancy, but not during the first trimester. Bacterial infections, as demonstrated by other antibiotic use and hospital admissions, were exceptionally assessed during the first trimester.

© 2024 Nakitanda AO et al. *JAMA Network Open.*
