# Supplementary material for: First Trimester Tetracycline Exposure and Risk of Major Congenital Malformations
Source: JAMA Netw Open. 2024 Nov 14;7(11):e2445055. doi: 10.1001/jamanetworkopen.2024.45055 (PMC11565264; doi:10.1001/jamanetworkopen.2024.45055)
Supplement: Supplement 2. — Data Sharing Statement [file jamanetwopen-e2445055-s002.pdf]

Data Sharing Statement

Nakitanda. First Trimester Tetracycline Exposure and Risk of Major Congenital Malformations. *JAMA Netw Open*. Published November 14, 2024. doi:10.1001/jamanetworkopen.2024.45055

Data
Data available: No

Additional Information
Explanation for why data not available: Sensitive personal data cannot be made available as mandated by data privacy laws in Sweden.
